# Repertoire of plant RING E3 ubiquitin ligases revisited: New groups counting gene families and single genes

**DOI:** 10.1371/journal.pone.0203442

**Published:** 2018-08-31

**Authors:** Domingo Jiménez-López, Francisco Muñóz-Belman, Juan Manuel González-Prieto, Victor Aguilar-Hernández, Plinio Guzmán

**Affiliations:** 1 Departamento de Ingeniería Genética, Centro de Investigación y de Estudios Avanzados del IPN, Unidad Irapuato, Irapuato, Gto., México; 2 Biotecnología Vegetal, Centro de Biotecnología Genómica, Instituto Politécnico Nacional, Reynosa, Tamaulipas, México; 3 CONACYT, Unidad de Bioquímica y Biología Molecular de Plantas, Centro de Investigación Científica de Yucatán, Col. Chuburná de Hidalgo, Mérida, Yucatán, México; George Washington University, UNITED STATES

## Abstract

E3 ubiquitin ligases of the ubiquitin proteasome system (UPS) mediate recognition of substrates and later transfer the ubiquitin (Ub). They are the most expanded components of the system. The Really Interesting New Gene (RING) domain contains 40–60 residues that are highly represented among E3 ubiquitin ligases. The *Arabidopsis thaliana* E3 ubiquitin ligases with a RING finger primarily contain RING-HC or RING-H2 type domains or less frequently RING-v, RING-C2, RING-D, RING-S/T and RING-G type domains. Our previous work on three E3 ubiquitin ligase families with a RING-H2 type domain, ATL, BTL, and CTL, suggested that a phylogenetic distribution based on the RING domain allowed for the creation a catalog of known domains or unknown conserved motifs. This work provided a useful and comprehensive view of particular families of RING E3 ubiquitin ligases. We updated the annotation of *A*. *thaliana* RING proteins and surveyed RING proteins from 30 species across eukaryotes. Based on domain architecture profile of the *A*. *thaliana* proteins, we catalogued 4711 RING finger proteins into 107 groups, including 66 previously described gene families or single genes and 36 novel families or undescribed genes. Forty-four groups were specific to a plant lineage while 41 groups consisted of proteins found in all eukaryotic species. Our present study updates the current classification of plant RING finger proteins and reiterates the importance of these proteins in plant growth and adaptation.

## Introduction

Protein modifications result in a diversification of their function. A 76-residue protein known as ubiquitin (Ub) is a post-translational protein modifier common to all eukaryotes that influences protein fate in several ways. Ub is a component of the ubiquitin proteasome system (UPS) wherein the Ub is transferred by three consecutive multi-enzymatic reactions E1-E2-E3 (E1, ubiquitin activating; E2, ubiquitin conjugating; and E3, ubiquitin ligase) to target the cellular protein repertoire. An array of Ub-dependent targets are degraded by the 26S proteasome, including receptors, transcription factors, metabolic enzymes and misfolded proteins which unveils the compelling function of ubiquitination. The UPS has been implemented by plants for development and response to environment cues [1, 2]. Non-degradative pathways result in either the activation of key signaling factors, endocytosis, or in the control of gene expression by modifying histone proteins [3]. Specific types of Ub modification govern the fate of ubiquitinated target proteins. For instance, monoubiquitination promotes endocytosis, Lys6-linked poly-Ub chains serve to control DNA repair processes (mainly Lys48-linked but also Lys11-linked poly-Ub chains degraded by the 26S proteasome), and Lys63-linked poly-Ub chains serve in protein activation [4, 5].

As the most expanded UPS component, E3 ubiquitin ligase families are the major source for precise recognition and Ub attachment. The different types of E3 Ub ligases are defined by the Really Interesting New Gene (RING) domain and a signature consisting of 40–60 residues delineated as Cys-X_2_-Cys-X_(9–39)_-Cys-X_(1–3)_-His-X_(2–3)_-Cys/His-X_2_- Cys-X_(4–48)_-Cys-X_2_-Cys, wherein Cys and His are metal ligands that bind two Zinc atoms and X is any amino acid residue [6, 7]. Two decades after discovering the RING finger domain, a vast array of proteins from diverse eukaryotes have been identified and revealed that the RING finger domain is often associated with other domains in the same molecule. This has been a convenient feature for classifying RING finger proteins based on their domain architecture [8–11].

A widespread survey identified the repertoire of RING finger proteins in *A*. *thaliana* [10]. RING finger domains were classified in two major types (RING-HC and RING-H2) and five minor types (RING-v, RING-C2, RING-D, RING-S/T and RING-G), wherein 30 groups were determined according to domain architecture [10]. The repertoire of RING finger proteins in other members of Viridiplantae, such as *Brassica rapa*, *Oryza sativa*, *Malus domestica*, and *Ostreococus tauri*, has been identified based on this classification [12–15]. We previously identified three families of RING finger type E3 ligases that encoded distinct motifs outside the RING domain. The first family is the *Arabidopsis* Tóxicos en Levadura (ATLs) which is plant specific [16]. ATL was named after the toxic phenotype was exhibited by the first member of the family, ATL2, when conditionally expressed in the yeast *Saccharomyces cerevisiae*. After testing 25 additional *A*. *thaliana* ATLs, only *ATL63* was shown to share the toxic phenotype in yeast [17, 18]. A common feature of ATLs is the presence of transmembrane helices at the amino-terminus that possibly drive the localization of ATLs to the plasma membrane and/or lipid rafts [19]. An overview of the ATL family from grape and tomato was recently reported [20, 21]. The second family is the BZF ATLs (BTLs) that encode a Ub binding domain at the amino terminal, C2/C2 Zinc Finger (BZF) [22]. The third family is the CTL that is identified by a motif named YEELL of unknown function [23].

ATL, BTL, and CTL are multiple gene families that comprise 91, 17 and 19 members, respectively. Curiously, a previous genome-wide classification of RING finger proteins into groups, based on domains outside the RING domain, placed members of these three families not in exclusive groups but each one into more than one group [10]. We noticed that the domain description in four of the thirty previously established groups was broad and general (no identifiable domains, coiled-coil, transmembrane domains, and signal peptide) and included more than 50% of the RING finger proteins [10]. We reasoned that ATL, BTL, and CTL proteins might be included in some of these groups. To further catalog RING finger proteins in plants and update their annotation, we surveyed RING fingers from protein databases derived from 30 species. We found that members of the ATL family belonged to two previously described groups while members of the BTL family belonged to three previously described groups. In addition, we arranged previously described families and single genes into 47 and 17 groups, respectively; and novel families and single genes into 19 and 17 groups, respectively.

## Results

### Identification and classification of the RING finger protein repertoire in *A*. *thaliana*

**Fig 1:**
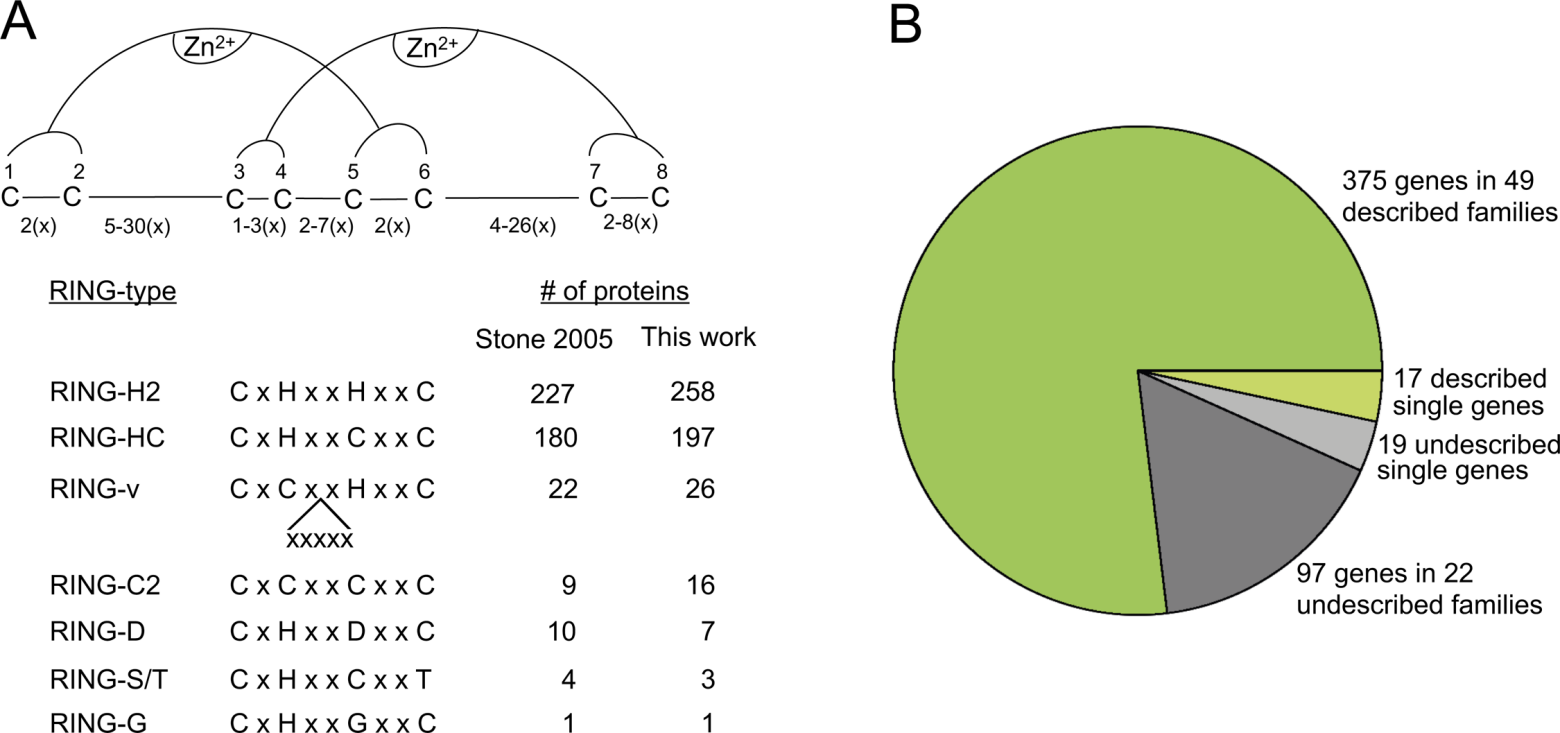
Types of RING finger domains and number of RING finger subfamilies in *A*. *thaliana*. A, Schematic representation of a canonical RING finger domain shows the eight residues involved in zinc coordination as cysteine (C) residues numbered from one to eight as well as the conventional number of amino acids represented by an x between them. Between the third and fourth zinc ligands, one to three residues were considered for all types of RING fingers. Between the fourth and fifth zinc ligands, two to three residues were considered for RING-H2, RING-HC, RING-D and RING-S/T, four to five residues for RING-C2 and seven residues for RING-v. Zinc ligand substitutions C>H, C>D, C>S, C>T or C>G were taken into account. For each RING-type, a consensus and the number of retrieved proteins are shown; data from Stone *et al*., 2005 was taken from S1 Table. B, Number of genes arranged in families or as a single gene is displayed. Described genes are shown in green and undescribed genes are shown in gray. Described families implied that there is a citation for at least one gene in the family (Table 2).

Hundreds of RING finger E3 ligases are predicted to be encoded by every plant genome that is currently available. Most of them are assigned to two main classes, RING-H2 and RING-HC, and the remainder are allocated to five less abundant classes. These RING finger classes may have one or more substitutions on the residues involved in zinc ligation and/or may differ in the spacing between the zinc ligands. We have previously based our survey of ATL, BTL, and CTL RING finger protein families on the layout of residues located at the RING domain followed by the inspection of transmembrane helices, YELL, or the GLD conserved motif. We noticed that the central region encompassing the third to sixth residues involved in zinc binding pinpointed particular subfamilies of RING finger proteins [23, 24]. We applied this reasoning to detect a comprehensive repertoire of *A*. *thaliana* RING finger proteins. We collected RING finger proteins by querying an array of up to 13 amino acids long patterns with a variable number of residues between the third and fourth zinc ligands as well as between fourth and fifth ligands. We then verified the presence of a RING finger in collected proteins and confirmed the annotation (Fig 1A) (see Material and Methods).

We ended up with 508 non-redundant *A*. *thaliana* RING finger proteins that were classified into the seven previously described RING-types. A comparison with the previous analysis indicated that 55 additional proteins were identified in our present analysis which corresponded to novel RING proteins (S1 Table). This rise in the number of protein-coding genes was expected due to the improved annotation of the *A*. *thaliana* genome (26,207 protein-coding genes in the TIGR 5.0 genome annotation, to 27,655 protein-coding genes in of current Araport11 genome annotation; https://www.arabidopsis.org/ and https://www.araport.org/, respectively). Thirteen proteins that were previously selected as RING finger proteins did not appear in our survey. An inspection of those RING proteins showed that ten of them were obsolete or incorrectly annotated loci and/or changed in numeral nomenclature while three of them encoded cysteine-rich regions that might not correspond to RING finger domains (red and gray shadowed cells, respectively, in S1 Table). These aforementioned RING proteins were discarded from our analysis. Three proteins formerly listed as RING-HC type domains were now found to match with a RING-H2 type domain (green shadowed cells in S1 Table).

As expected in our survey, two major types of proteins were detected corresponding to RING-H2 and RING-HC as well as members of the previously described RING finger types, RING-v, RING-C2, RING-D, RING-S/T, and RING-G (Fig 1A and S1 Table). Since several RING genes expanded as gene families in the plant genomes, we identified single genes based on all-against-all BlastP searches. For the single genes in *A*. *thaliana*, we used the following acronyms: LHC for Lone RING-HC type protein, LHH for Lone RING-H2 type protein, Lv for Lone RING-v type protein, and LCC for Lone RING-C2 (S2 Table). A total of 36 RING genes were identified as single genes and corresponded to 7% of the total RINGs identified. We inspected the annotation of those RINGs and found that 19 RINGs were undescribed single genes. Among the 17 RINGs described single genes, some played a role in photomorphogenesis (LHC01 and LHC08), abiotic stress (LHC02, LHH05, and LHH06), or in the protein complex APC responsible for controlling the cell cycle (LHH01) (Table 1). Besides the single RING genes, we classified RING protein families that had at least two putative orthologs related by the sequence outside the RING domain. To organize the repertoire of RING proteins, we used our previous nomenclature of ATL, BTL, and CTL for the new RING-H2 and RING-HC families (see S2 Table). We modified our ATL acronym interpretation from “*Arabidopsis* Tóxicos en Levadura” to: ATL, A-Type RING Ligase family. The RING finger protein families were named as follows: DTL, D-Type RING Ligase family; ETL, E-Type RING Ligase family, and so on, up to UTL, U-Type Ligase family. We applied a code to assist in the handling of previously described protein families as follows: FHC01 up to FHC22 for RING-HC families and FHH01 up to FHH15 for RING-H2 type families. Similarly, for the RING-v and RING-C2 families we used Fv and FCC, respectively (see Tables 2 and S2). Ninety-three percent of the genes were members of gene families and 7% were single genes. We also considered annotation and citations to determine whether a gene or gene family was previously described with regards to domain structure or functional analysis (Table 1). We found that 80% of the genes were included in previously described gene families or single genes while 20% were previously undescribed genes (Fig 1B). Of the 107 established groups, 66 were arranged into previously described groups and 41 were arranged into previously undescribed groups (Fig 1B and S2 Table).

**Table 1:** Groups of “lone” Arabidopsis RING finger proteins.

Groups				
This work	Stone et al.	No of proteins	Description	References
LHC01	1	1	COP1 SUPPRESSOR 1 (CSU1)	[67]
LHC02		1	HIGH EXPRESSION OF OSMOTICALLY (HOS1)	[68]
LHC03	1	1	NO CATALASE ACTIVITY 1 (NCA1)	[69]
LHC04	2.2*	1	PROTEOLYSIS 1 (PRT1)	[70]
LHC05	4.1	1	serine/threonine phosphatase 6 regulatory ankyrin repeat	[10]
LHC06	4.2*	1	KEEP ON GOING (KEG)	[71]
LHC07	22*	1	RELATED TO KPC1 (RKP)	[72]
LHC08	28.1*	1	CONSTITUTIVE PHOTOMORPHOGENIC 1 (COP1)	[73]
LHC09	29.1	1	HAKAI	[74]
LHC10, LHC11, LHC15	1	1, 1, 1	undescribed	
LHC12, LHC13		1,1	undescribed	
LHC14	29.2	1	undescribed	
LHC16	14	1	undescribed	
LHC17	24	1	undescribed	
LHH01	1	1	ANAPHASE-PROMOTING COMPLEX (APC11)	[75]
LHH02	1	1	RWD domain-containing protein	[10]
LHH03		1	CALCIUM-DEPENDENT KINASE ADAPTER (CDPK)	[76]
LHH04		1	INCREASED DNA METHYLATION 1 (IDM1)	[77]
LHH05		1	ABI3-INTERACTING PROTEIN 2 (AIP2)	[78]
LHH06	24	1	SUGAR-INSENSITIVE 3 (SIS3)	[79]
LHH07, LHH08, LHH09, LHH10, LHH11	1	1, 1 1, 1, 1	undescribed	
LHH12, LHH13		1, 1	undescribed	
LHH14	6	1	undescribed	
LHH15	24	1	undescribed	
LCC01		1	GENERAL TRANSCRIPTION FACTOR II H2, (GTF2H2)	[80]
LCC02		1	FYVE-DOMAIN PROTEIN 1, (FYVE1/FREE1)	[81]
Lv01	1	1	undescribed	
Lv02	24	1	undescribed	

* concordance with Stone *et al*., 2005 groups. Stone *et al*., 2005 groups 1, 6, 24 and 25 are shadowed.

**Table 2:** Groups of Arabidopsis RING finger protein families.

Groups				
This work	Stone et al.	No of proteins	Description	References
FHC01	11.1*	38	RING BETWEEN RING FINGERS (RBR)	[82]
FHC02	21*	14	TNF RECEPTOR-ASSOCIATED FACTOR (TRAF)-like	[83]
FHC03	8.1*	12	SNF2-Helicase	[84]
FHC04	1,6	11	BOTRYTIS SUSCEPTIBLE (BOI)	[38]
FHC05		10	SHORT INTERNODES/ STYLISH (SHI/STY)	[85]
FHC06	1,6,24	9	RING MEMBRANE-ANCHOR (RMA)	[86]
FHC07	26*	6	ORTHRUS/VARIANT IN METHYLATION (ORTH/VIM)	[87]
FHC08	1	5	ABA INSENSITIVE RING PROTEIN (AtAIRP4)	[88]
FHC09	27.2*	5	RING DOMAIN LIGASE (RGLG)	[89]
FHC10	4.1	5	XB3 ORTHOLOG 2 (XBAT32)	[90]
FHC11	24	5	ABERRANT POLLEN DEVELOPMENT (APD)	[91]
FHC12	1,24	5	LOSS OF GDU 2 (LOG2), LUL, AIRP3	[92]
FHC13	1	3	DREB2A-INTERACTING PROTEIN	[93]
FHC14	24,25	3	Transmembrane Fragile-X-F-associated protein	[10]
FHC15	1,24	3	SUPPRESSOR OF PLASTID PROTEIN IMPORT 1 (SP1)	[42]
FHC16	23*	2	BENZOIC ACID HYPERSENSITIVE1-DOMINANT(BAH1)	[94]
FHC17	1,6	2	RING 1	[95]
FHC18	5*	2	BRCA1/BARD1	[96]
FHC19	1,6	2	HISTONE MONO-UBIQUITINATION (HUB)	[97]
FHC20	18*	2	PEROXIN	[98]
FHC21	8.2*	2	helicase domain-/ IBR domain-containing protein	[10]
FHC22	13*	2	KINESIN 7	[99]
FHH01	1,24,5	9	XERICO/BRH1	[51]
FHH02	27.1*	8	von Willebrand factor type A (VWA)	[100]
FHH03	1,9,24	7	BRUTUS (BTS)	[101]
FHH04	16*	6	RECEPTOR MEMBRANE RING-H2 (RMR)	[102]
FHH05	1	5	AIRP1/RHB1A	[103]
FHH06	1	4	RFI2	[104]
FHH07	25	2	RHA2	[105]
FHH08	24	2	HDR1	[106]
FHH09	24	2	FLYING SAUCER (FLY1)	[107]
FHH10	29.3*	2	BRAP2 RING ZNF UBP DOMAIN (BRIZ)	[108]
FHH11	1	2	ROC1	[109]
FHH12	1	2	RING-H2 group F 1 (RHF1)	[110]
FHH13	7*	2	RPM1 INTERACTING PROTEINS (RIN)	[53]
FHH14	1,24	2	SALT- AND DROUGHT-INDUCED RING FINGER1 (SDIR1)	[60]
FHH15	6, 17.2	2	VACUOLAR PROTEIN SORTING	[111]
FCC01	12*	6	Transcription factor jumonji	[112]
FCC02	20*	3	RNA binding (RRM/RBD/RNP motifs)	[10]
FCC03		2	RING FYVE domain proteins	[113]
FCC04	1	3	undescribed	
Fv01	1,24	8	AtRZFP	[114]
Fv02	24	2	SHOOT APICAL MERISTEM ARREST (SHA1)	[115]
Fv03	1,24	2	ECERIFERUM9 (CER9)	[116]
Fv04	24	5	undescribed	
Fv05, Fv06	1,24	4, 3	undescribed	
FD	1	7	RING-D	[10]
FG	6	1	Transducing/WD40 repeat-like	[10]
FS/T	15*	3	LisH/CRA/RING	[10]
ATLs	24,25	100	Arabidopsis tóxicos en levadura	[16]
BTLs	1,24, 30	17	BZF ATLs	[22]
CTLs	1	19	C-type ATLs	[23]
DTL, ETL, HTL, ITL, JTL, LTL, QTL, RTL, STL	1	11, 12, 5, 2, 2, 6, 2, 2, 2	undescribed	
FTL	24	10	undescribed	
GTL, TTL	1,6	6, 4	undescribed	
KTL, OTL, PTL	24,25	2, 3, 3	undescribed	
MTL	3, 6	4	undescribed	
NTL	6, 29.2	3	undescribed	
UTL	29.1	2	undescribed	

* concordance with Stone *et al*., 2005 groups. Stone *et al*., 2005 groups 1, 6, 24 and 25 are shadowed.

We contrasted our collection with a comprehensive catalog of RING proteins from *A*. *thaliana* that classified 468 RING proteins into 32 different groups. We encountered all of the established groups and a vis à vis concordance with 21 of them (Tables 1 and 2, groups denoted with an asterisk). These groups included distinct domains besides the RING domain (see the RING BETWEEN RING FINGERS, the TRAF-like and the SNF2-Helicase families encoding RING-HC domain, the first three groups in Table 2). A discrepancy was evident with 296 RING proteins that were formerly classified as either encoding no identifiable domain, a coiled coil domain, transmembrane domains, or a signal peptide. These RING proteins belonged to the previously established groups 1, 6, 24 and 25, respectively, and were distributed in our classification among 31 new groups (see Tables 1 and 2). For instance, members of the ATL family were placed in groups 24 and 25; members of the BTL family in groups 1, 24, and 25; and members of the CTL family in group 1 (see Table 2). Among the 41 undescribed groups (families and lone genes), we found that 34 of them were placed in four previously established groups: 1, 6, 24 and 25 (shadowed in Tables 1 and 2). Thus, they could be further catalogued.

### Phylogenetic relationships of *A*. *thaliana* RING-HC and RING-H2 protein groups

**Fig 2:**
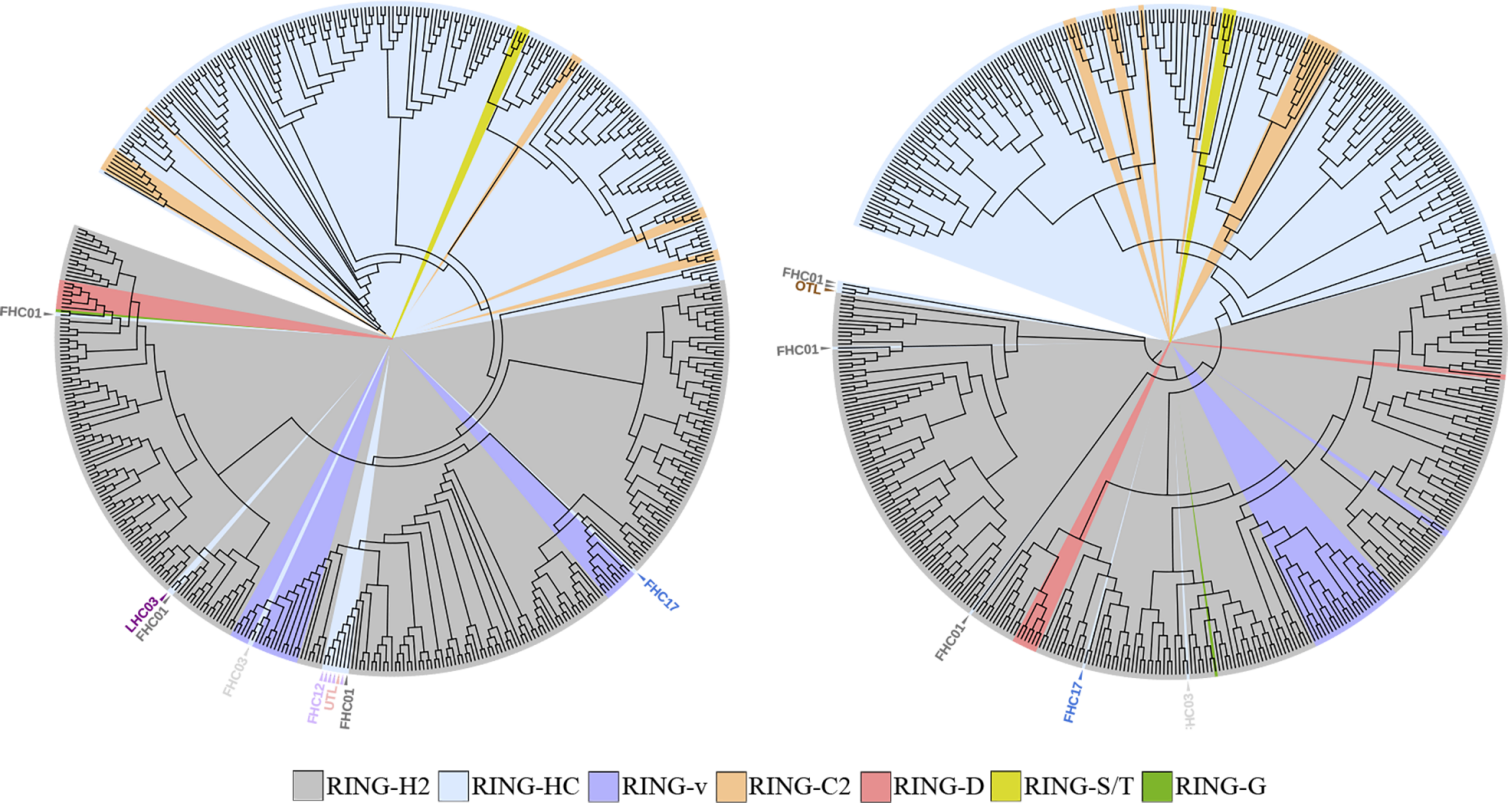
Phylogenetic distribution of *A*. *thaliana* RING finger proteins within seven RING domains. The tree was generated with the RING finger domain from 508 RING finger polypeptide sequences of *A*. *thaliana* by the NJ (left) or ML (right) methods. RING-H2 and RING-HC domains are highlighted in gray and blue shades, respectively. Color codes highlighting RING-v, RING-C2, RING-D, RING-S/T, and RING-G are indicated. Misplaced sequences are denoted with arrowheads followed by the family code name. From proteins that contain two RING domains, the sequence from the domain that is present in all members of the family was used to build the tree (12 out the 38 RING sequences from FCH01, five out the six RING sequences from FCH07, one RING sequence from both LHC01 and LHC04; see S1 Table).

To support the classification of RING proteins, we first analyzed phylogenies of the *A*. *thaliana* repertoire based on the sequences of the RING domains to find similar sections within RING proteins. We compared phylogenies obtained with the neighbor-joining (NJ) and the maximum-likelihood (ML) methods that were based on 508 RING domains. As previously inferred, RING domains from most ATL, BTL, or CTL proteins had enough sequence-specific determinants to sustain clustering. We found that both NJ and ML methods showed resolution of the seven RING finger-types, grouping most of the RING-HC and RING-H2 domains in two separate clades (blue and gray colored branches, respectively, in Fig 2). In both trees, RING-D, RING-G and RING-v domains were contained in the RING-H2 clade while the RING-C2 and RING-S/T domains were in the RING-HC clade (Fig 2). These data suggested that the variants RING-D, RING-G and RING-v were evolutionarily related to the RING-H2 domain while the RING-C2 and RING-S/T were related to the RING-HC domain. Few misplaced sequences were observed in these trees. Three members of FHC01, one of FHC03, and one of FHC17 encompassed RING-HC and appeared among the RING-H2 clade in both trees. The FHC12 group, one UTL member, and LHC03 were only found in the NJ tree (see Fig 2, left panel). Likewise, one OTL member appeared among the RING-H2 clade only in the ML tree (see Fig 2, right panel).

**Fig 3:**
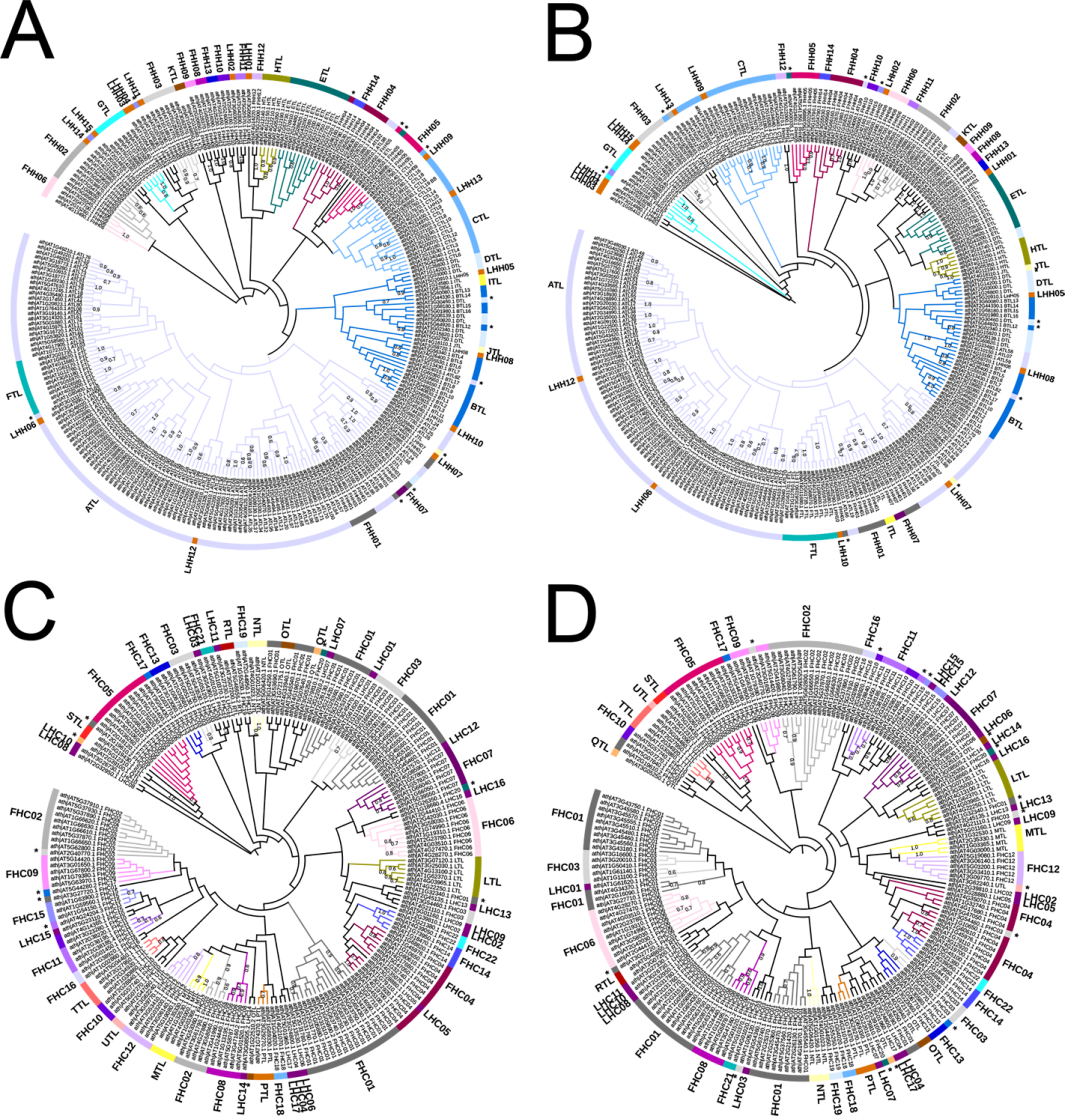
Phylogeny of *A*. *thaliana* RING-HC and RING-H2 finger domains. Trees are displayed that were generated by the NJ method (A, C) and the ML method (B, D) from 258 *A*. *thaliana* RING-H2 (A, B) and 197 RING-HC domain sequences (C, D). A wide range of colors were used to distinguish each family. Misplaced sequence are denoted with arrowheads followed by the family name.

We then examined the phylogenetic distribution of *A*. *thaliana* RING finger proteins in two separate trees generated by both NJ and ML methods. One tree was based on 258 RING-H2 domains and the other on 197 RING-HC domains (Fig 3). The overall phylogenetic relationship among RING finger domains showed a topology that was coherent with the distribution of the predicted families, particularly in families with few members. The tree including RING-H2 domains consisted of one major clade containing most ATLs and six to nine minor clades that included the additional groups (Fig 3A and 3B). Clades of ATLs, BTLs, and CTLs—the largest RING-H2 families—comprised of other families or single genes that lacked common features found within ATLs, BTLs and CTLs. For instance, the ATL clade in both NJ and ML trees harbored the families FTL, FHH01, and FHH07 as well as the single genes LHH06, LHH07, LHH10, and LHH12 (see light violet clade in Fig 3A and 3B). The BTL and DTLs were both included in the same clade together with JTLs, LHH08, and LHH05 (see blue clade in Fig 3A and 3B). CTLs included LHH09 and LHH13. The presence of more than one group within the same clade indicated that these proteins might have preserved a common RING-type and acquired diverse domains or conserved motifs outside the RING finger domain as an accessory sequence that could assist to their proper function. Indeed, BTLs and DTLs contain a highly similar RING finger but only BTLs contain an Ub binding domain BZF (see below). On the other hand, the tree including RING-HC groups showed the dispersion of the FHC01 and FHC03 families in at least three clades (FHC01 NJ tree and FHC03 NJ and ML trees, Fig 3C and 3B).

### Constraint on the RING domain in RING families

**Fig 4:**
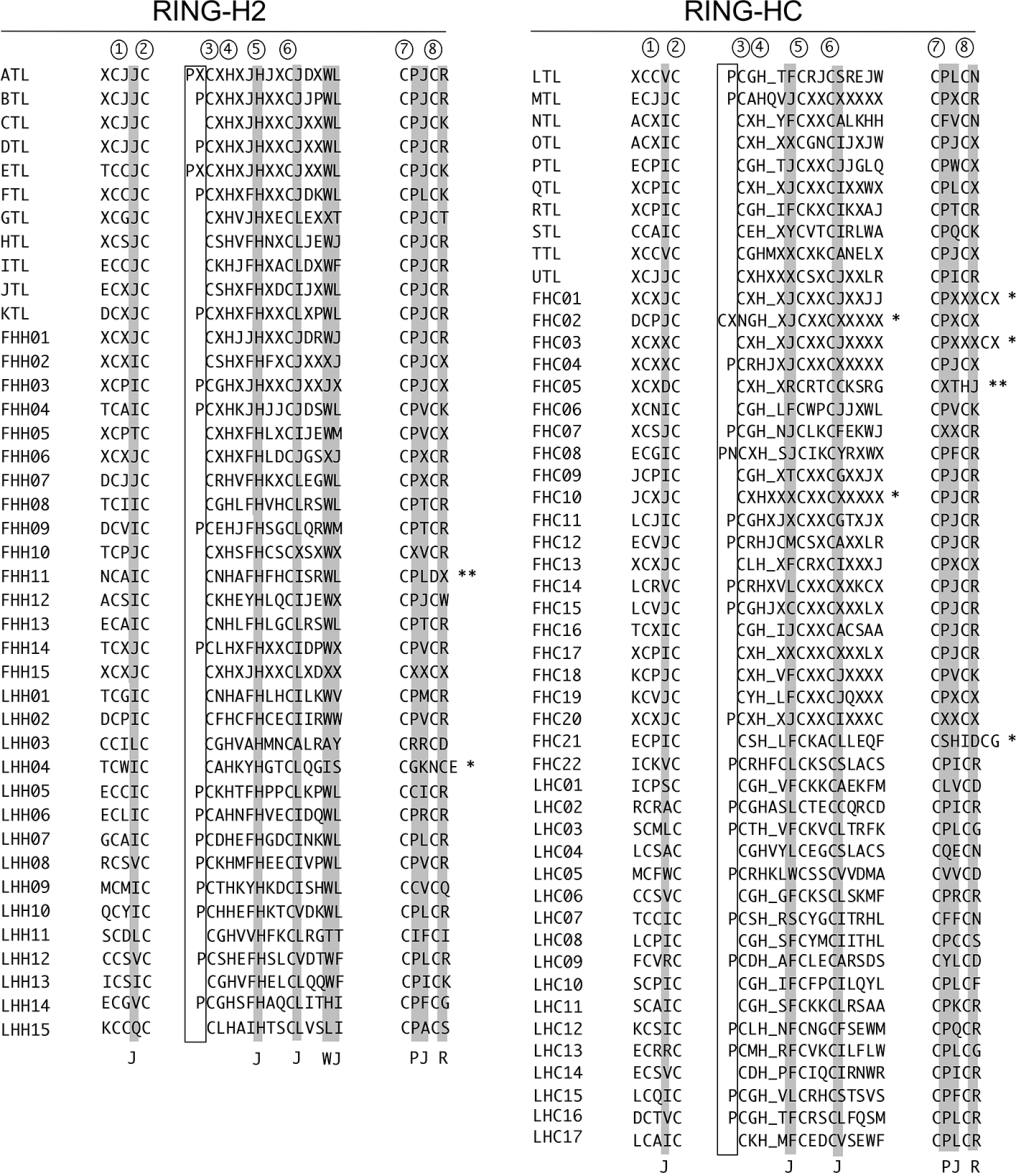
RING finger domains on *A*. *thaliana* groups. Three regions of multiple consensus sequences alignment from the 41 RING-H2 or the 49 RING-HC groups that encompass the residues involved in zinc ligation are displayed. The circled numbers indicate the residues involved in zinc ligation. The amino acid length of three regions from the RING finger domain is shown: five residues encompassing ligands 1 and 2; 14 to 17 residues from the central region encompassing ligands 3, 4, 5 and 6; and five residues encompassing ligands 7 and 8. The shaded blocks indicate common residues to most RING fingers; J corresponds to any hydrophobic residue. The location of proline residues previously described in ATL and BTL families is enclosed by a rectangle. The symbol * denotes regions that deviate in length from the canonical sequence (FHC01, two extra residues; FHC02, two extra residues; FHC03, two extra residues in 50% of the proteins; FCH10, three proteins show the canonical distances and two proteins show an extra residue between the 4^th^ histidine and 5^th^ cysteine; FHC21, two extra residues). The symbol ** represents the alignment denoting a substitution of one of the zinc ligation residues in all members of the family (FHH11, C to D; FHC05, C to H). The underscore indicates a gap within the alignment.

An alignment of consensus sequences neighboring the residues involved in zinc ligation RING finger sequences from the 59 families showed that five residues were present in most of the RING finger domains (three hydrophobic residues, a proline and an arginine in shadow blocks; Fig 4). Three residues were more conserved in RING-H2 than in RING-HC families, including two hydrophobic residues and a tryptophan in the central region of the domain (shadow blocks in RING-H2 sequences only, Fig 4). The highly conserved proline residue adjacent to the third cysteine in the RING domain that was common in both ATLs and BTLs was present in several groups. This proline in ATLs was one residue from the third cysteine and was found in this location in only two families (ETL and FHC08, Fig 4). Similarly in BTLs, 18 families carried a proline adjacent to the third cysteine (enclosed by a rectangle, Fig 4). As suggested for ATLs and BTLs, there was functional constraint on the arrangement of this proline in the RING domain of several families. Length deviations from the canonical sequence were only detected in RING-HC domains. A single additional residue that was commonly observed in RING fingers between the fourth and fifth occurred in ten families (MTL, TTL, UTL, FCH04, FHC10, FCH11, FCH12, FCH14, FCH15, FCH22, Fig 4). The family FHC02 included an extra residue between the third and fourth residues, and three families included two extra residues between the seventh and eighth residues (FHC01, FHC03, FHC21; Fig 4). Moreover, substitution of the zinc ligation residue was observed in two families within the eighth residue, including changing to aspartic acid in FHH11 and changing to histidine in FHC05 (Fig 4).

### Gene architecture of RING finger genes

Examination of gene architecture in ATL, BTL, and CTL genes indicated that spliceosomal introns within the coding DNA sequence (CDS) was a distinctly conserved feature for each family. Spliceosomal introns in ATL and BTL genes were not present in nearly 90% of the genes, whereas CTL genes generally contained between three and six introns. Similarly, introns in the 5’ untranslated region (UTR) that impacted gene expression were also a distinctly conserved feature among these three gene families. Spliceosomal introns within the 5' UTR were detected in a number of plant BTL and CTL genes but not in ATL genes. To further support the classification of RING finger proteins through the information on gene structure, we inspected the presence of spliceosomal introns within the CDS and the 5' UTR in genes of two angiosperms: the eudicot *A*. *thaliana* and the monocot *O*. *sativa* (see Table 3). Except for seven families (FHC01, FHC03, FHC10, FHH03, FHH15, FS/T, and CTL) that showed a wide interval range (dispersion of CDS introns greater than two times, Table 3), we observed a minor variation in the number of introns within the families indicating that gene architecture was preserved in most families. When comparing *A*. *thaliana* and *O*. *sativa*, we observed a discrepancy in about 10% of the groups (FHC11, FHH02, FG, FS/T, ETL, TTL, LHC07, LHC16, LHH03; shadowed rows in Table 3), suggesting evolutionary conservation of gene structure in most groups. Thirteen percent of *A*. *thaliana* groups and 19% of *O*. *sativa* included at least one intron at the 5’ UTR. We detected this co-occurrence in six of the groups (FHC06, FHC16, BTLs, CTL, GTL, and PTL), suggesting that this intron was an evolutionarily conserved feature in these groups.

**Table 3:** Incidence of spliceosomal introns in genes of RING groups.

	CDS introns (range)		5'UTR intron (occurrence)	
Group	*A*. *thaliana*	*O*. *sativa*	*A*. *thaliana*	*O*. *sativa*
FHC06, BTLs, PTL	0, 0, 0	0, 0, 0	Yes	Yes
FHC16	5	5	Yes	Yes
Fv02	2	2	Yes	
DTLs	0	0		Yes
KTLs, MTLs, FTLs, FHH14	2, 1, 2–4, 7	2, 1 2–4, 7		Yes
FHH01, FHH07	0, 0	0, 0		
ATLs, FHC01	0–3, 0–14	0–3, 0–14		
FHC05, Fv05, LHC01	1, 1, 1	1,1, 1		
FHC12, VTLs	2, 2	2, 2		
LCC01	3	3		
FHH11, LHH05	4, 4	4, 4		
LHC14, LHH10	5, 5	5, 5		
LHC03, LHH02	6, 6	6, 6		
Fv03, LHC10, LHH13, LHH15	8, 8, 8, 8	8, 8, 8, 8		
LHC02, LHH06	9, 9	9, 9		
FHC04, FHC15, FHC20	1–3, 5–10, 7–10	1–3, 5–10, 7–10		
FHH15	4–18	4–18		
LHC08, FHC14, FHC19, LHH14	12, 13, 18, 22	12,13, 18, 22		
CTLs	0–8	3–6	Yes	Yes
GTLs	1–3	2	Yes	Yes
Fv01	5–6	6–7	Yes	Yes
NTLs	2	1	Yes	
FS/T, ETLs	0, 0	0–6, 4		Yes
LHH01	2	0–1		Yes
RTLs, FHH05	3, 3	3–4, 3–4		Yes
FHH04	1–4	3		Yes
FHH08	5–6	6		Yes
FHH12	7	6		Yes
LHC07	9	4		Yes
FHC21, LHC09	3, 3	1, 2		
LHH03	3	6		
UTLs	6	4–6		
FG	6	11–12		
LHH04	7	7–9		
LHC04, LHC17	8, 8	7, 7		
LCC02	9	8		
FHC11	9	4		
FHC17	9	9–13		
FHH10, FHH13	10, 11	11–12, 6–11		
LHC12, LHC15	13, 12	15, 10		
LHC16	16	5		
FHC22	24	23		
LTLs, FHH02	0–1, 0–1	0, 0–4		
FHC02	1–2	1–3		
FHH06	2–5	5		
Fv06	2–6	5–6		
FHC03	2–18	2–19		
FHC08, FCC03	3–4, 3–4	3–6, 4		
QTLs	4–6	5		
FHC13	5–6	6		
TTLs	5–6	6–11		
Fv04	5–7	4–8		
FHC07, FHC10, FHC18	6–8, 7–9, 12–13	8, 0–10, 13		
FCC01, FCC02	7–12, 11–12	4–15, 7–11		
FHH03, FHH09	11–13, 12–13	0–13, 13		

Gene structure was not available for the following *O*. *sativa* groups: FHC09, FD, HTLs,

ITLs, JTLs, OTLs, STLs, LHC05, LHC06, LHC11, LHC13, LHH07, LHH08, LHH09,

LHH11, LHH12, Lv01, Lv02.

### Distinctive domain architecture for each RING finger group

We obtained a general domain architecture view of the RING finger protein groups by conducting conserved motif searches with the MEME server that provided a catalog of LOGOs as output. We performed separate searches for each one of the six types of RING finger proteins. Among the different types of proteins, the RING domain commonly encompassed between two to four sequence LOGOs which were common to most of the proteins of its type (data not shown). In an attempt to typify and distinguish each one of the RING finger group individually, we selected up to three distinct sequence LOGOs that distinguish a group within the RING proteins identified. A synopsis of the mapped LOGOs is presented in Table 4, and the sequence LOGO catalog is displayed in S3 Table. A unique domain structure was predicted for each group, providing reliable evidence for the proposed categorization of the RING protein superfamily. An exception was present for nine groups for which only sequence LOGOs encompassing the RING domain were generated (ITL, JTL, FHH07, LHH07, LHH08, LHH11, LHH12, LHC13 and Lv01; see Methods).

**Table 4:** Sequence LOGOs mapped to RING finger groups.

RING-H2		RING-HC		RING-C2	
Group	LOGO	Group	LOGO	Group	LOGO
ATL	(4*)	LTL	(114)	FCC01	(4,1,3)
BTL	(28,38,11**)	MTL	(61,64,63)	FCC02	(23,5,27)
CTL	(5)	NTL	(66)	FCC03	(10,13,41)
DTL	(11**)	OTL	(160)	FCC04	(47,93,46)
ETL	(13)	PTL	(118,106)	LCC01	(26,38,19)
FTL	(8,10,4*)	QTL	(145)	LCC02	(66,35,25)
GTL	(62)	RTL	(219)		
HTL	(23)	STL	(109,217)	**RING-v**	
KTL	(189)	TTL	(70)	Group	LOGO
FHH01	(65)	UTL	(75)	Fv01	(5,4,6)
FHH02	(12,36,14)	FHC01	(8)	Fv02	(15,16,13)
FHH03	(95,19)	FHC02	(21)	Fv03	(26,2,27)
FHH04	(97,15,25)	FHC03	(6,37,3)	Fv04	(11,3,7)
FHH05	(84)	FHC04	(71,15)	Fv05	(8)
FHH06	(174,160,191)	FHC05	(99,23)	Fv06	(14,9)
FHH08	(33,39)	FHC06	(132,174)	Lv02	(42,34,43)
FHH09	(55,80)	FHC07	(10,28)		
FHH10	(56)	FHC08	(60,49)		
FHH11	(103,195)	FHC09	(17,32,30)	**RING-D**	
FHH12	(113,143)	FHC10	(62)	Group	LOGO
FHH13	(85,54)	FHC11	(46,50,57)	FD	(2,3,4)
FHH14	(112,120,173***)	FHC12	(97,36)		
FHH15	(121)	FHC13	(173,111,188)	**RING-G**	
LHH01	(116)	FHC14	(65,83,84)	Group	LOGO
LHH02	(155)	FHC15	(124,122)	FG	(5,9,1)
LHH03	(157,165,175)	FHC16	(144,113,153)		
LHH04	(110,104,107)	FHC17	(117,147)	**RING-S/T**	
LHH05	(162,192,173***)	FHC18	(178,128,119)	Group	LOGO
LHH06	(154,105,117)	FHC19	(123,133,116)	FS/T	(4,3,2)
LHH09	(173***)	FHC20	(104)		
LHH10	(168,190,173***)	FHC21	(78,80,93)		
LHH13	(100,148)	FHC22	(81,89,87)		
LHH14	(135,193,126)	LHC01	(134,120,135)		
LHH15	(124,170,164)	LHC02	(184,195,155)		
		LHC03	(176)		
		LHC04	(187,152)		
		LHC05	(218,154)		
		LHC06	(182,165,166)		
		LHC07	(140,197,157)		
		LHC08	(115,172,151)		
		LHC09	(116,134,127)		
		LHC10	(175)		
		LHC11	(108,137,112)		
		LHC12	(120)		
		LHC14	(131,136,125)		
		LHC15	(186,164,185)		
		LHC16	(183)		
		LHC17	(105,170,109)		

LOGOs were generated from each type of RING finger proteins separately; numbers are sole for each type. *, ** and *** represent LOGOS that are common to more than one group. Sequence LOGO were not generated for the following groups: ITL, JTL, FHH07, LHH07, LHH08, LHH11, LHH12, LHC13 and Lv01.

**Fig 5:**
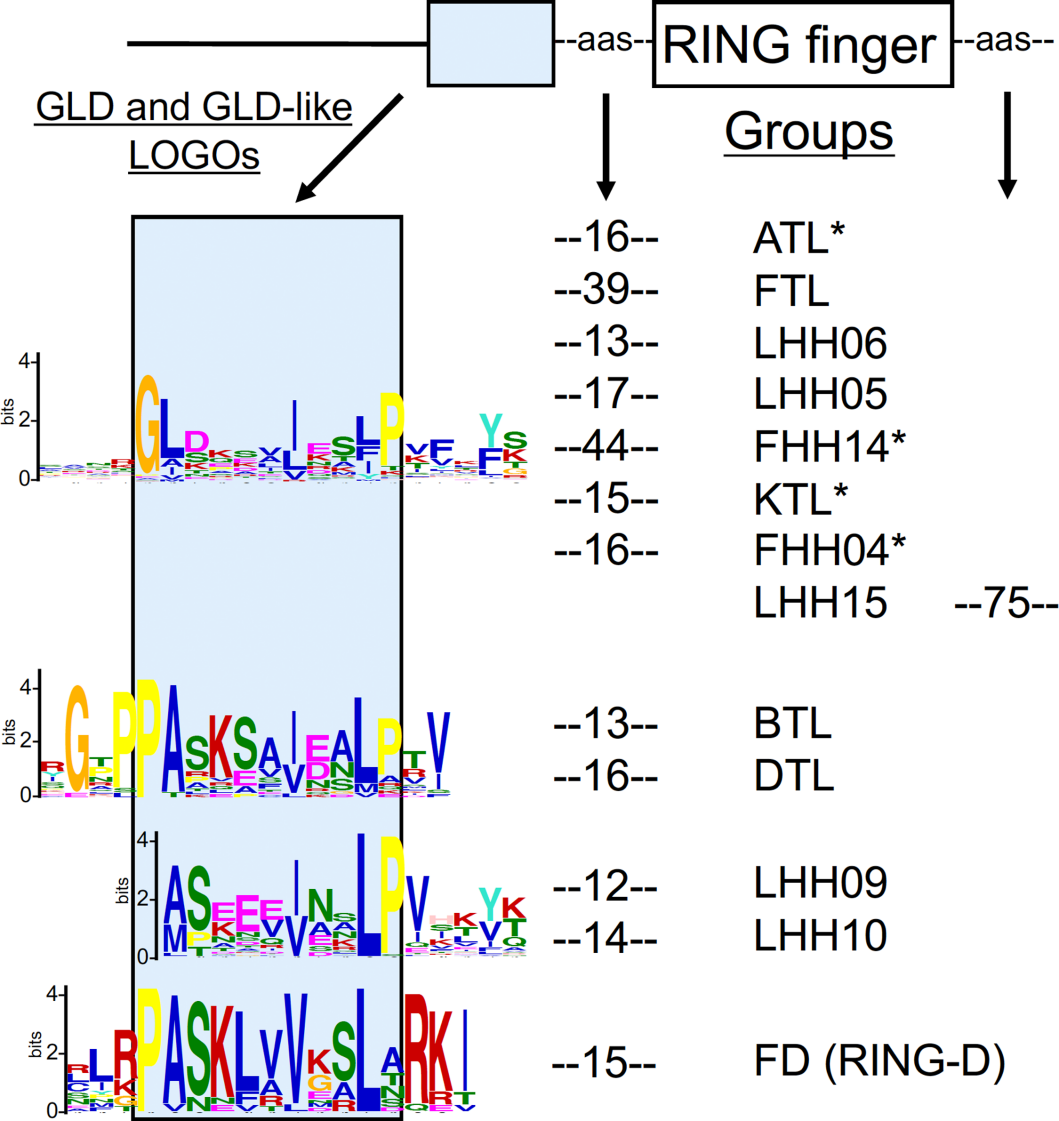
GLD and GLD-like LOGO sequences in *A*. *thaliana* RING finger proteins. An alignment of four sequence LOGOs that include the GLD and GLD-like conserved motifs obtained from RING-H2 or RING-D finger proteins (from top to bottom: LOGO #4, #11, #173, and #2 FD; see S3 Table). The location of sequence homology among LOGOs is highlighted by the blue square in the diagram representation. Except for Group LHH15, in all cases the LOGO sequences were located upstream and adjacent to the RING domain. The average distance between the LOGO and the start of the RING finger domain is presented in numbers of amino acids (aas). The symbol * depicts groups for which at least 50% of the members included sequence LOGO #4.

A few non-RING finger sequence LOGOs were found to be common in more than one group. A LOGO containing the GLD motif (RING-H2, LOGO #4) was present in ATLs and FTLs. The GLD motif was distinctiveness among ATLs and was generated in 85% of this group, in all nine members of FTL, and in 50% of the proteins from three additional groups (FHH14, KTL, and FHH04). Sequence LOGOs #11 and #173 from RING-H2 proteins were generated in two (BTL, DTL) and four groups (FHH13, FHH14, LHH05, and LHH09), respectively (Table 4). Inspection of RING-H2 LOGOs #4, #11 and #173 revealed a similarity among their primary sequences. Furthermore, the location of these three LOGOs relative to the RING-H2 domain match was located closely upstream to the RING finger domain. We inspected the other RING finger types for related sequence LOGOs and found that RING-D LOGO #2 was related in sequence and was also close to the RING domain. An alignment of these four sequence LOGOs is displayed in Fig 5. We further searched sequences for common LOGOs among RING finger types and did not detect any readily noticeable sequence that might be present in more than one type of protein. A similar result was inferred after searching for LOGO sequences generated from all RING finger proteins (data not shown).

### Prevalence of RING-types across eukaryotes

To gain an insight into the distribution of RING finger protein groups present in *A*. *thaliana*, among other eukaryotes, and to assess their evolutionary history, we surveyed representative proteomes across plants, animals, fungi, and protists. We performed BLAST searches using *A*. *thaliana* RING sequences as a query followed by an inspection of the LOGOs from each of the inferred groups. We surveyed the proteome of 29 eukaryotes, including 12 viridiplantae. The viridiplantae proteomes included two green algae (*Micromonas* sp. RCC299 and *Chlamydomonas reinhardtii*) and ten embryophytes (two basal land plants models: the moss *Physcomitrella patens* and the lycopod *Selaginella moellendorffii*, one basal angiosperm: the primitive magnoliophyte *Amborella trichopoda*, two monocots: *Zea mays* and *Oryza sativa*, and five eudicots: *Solanum lycopersicum*, *Medicago truncatula*, *Populus trichocarpa*, *Citrus sinensis* and *Arabidopsis lyrata*). The animal genomes consisted of three basal (the fresh-water polyp *Hydra vulgaris*, the placozoan *Trichoplax adhaerens* and the sponge *Amphimedon queenslandica)*, two basal vertebrates (the sea squirt *Ciona intestinalis* and the purple sea urchin *Strongylocentrotus purpuratus*), two invertebrates (the nematode *Caenorhabditis elegans* and the insect *Drosophila melanogaster*), six vertebrates (the fish *Danio rerio*, the amphibian *Xenopus laevis*, the reptile *Alligator sinensis*, the bird *Gallus gallus*, and the mammals *Mus musculus* and *Homo sapiens*). Two protist groups were surveyed, Kinetoplasts (*Trypanosoma cruzi*) and Apicomplexans (*Plasmodium vivax)* as well as two groups of fungi species, Eurotiomycetes (*Aspergillus niger*) and Saccharomycetes (*Saccharomyces cerevisiae*).

**Fig 6:**
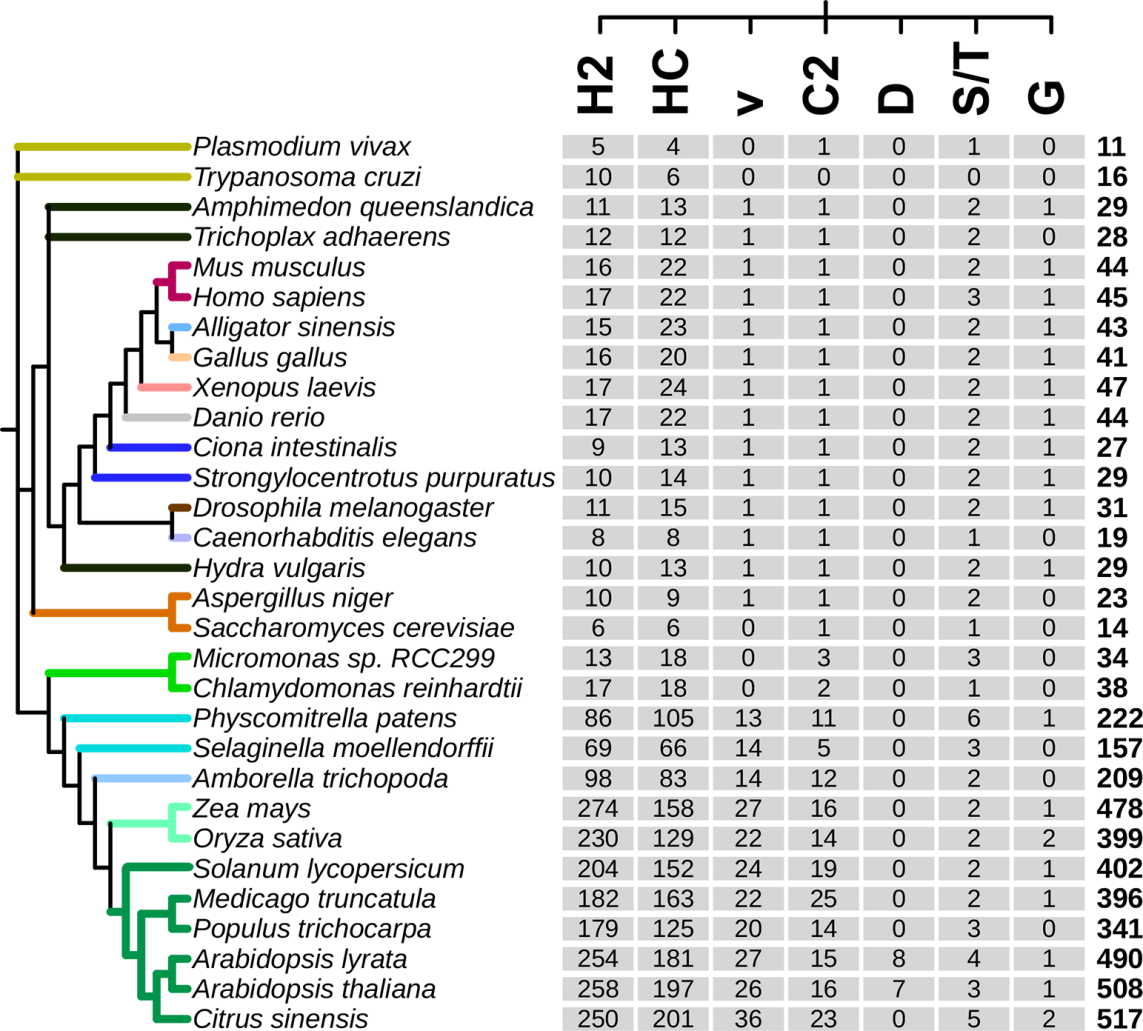
Number of retrieved RING-type proteins types across eukaryotes. The phylogenetic relationship between thirteen viridiplantae (two green algae and eleven embryophyta), thirteen animal (six vertebrates, three basal animals, two basal vertebrates, two invertebrates), two fungal, and two protist genomes was based on data from the National Center of Biotechnology Information (NCBI) taxonomy server (http://www.ncbi.nlm.nih.gov/Taxonomy). The color code for a selected group of organisms is displayed at the branches. The identifiers for each RING-type are displayed in S1 Table and a list of retrieved genes in S4 Table. The total number of RING finger proteins is shown at the last column.

We retrieved 4203 RING proteins from the 29 species and categorized them into each of the seven RING finger types established with 508 *A*. *thaliana* RING proteins (Fig 6). In all species, the RING-HC and RING-H2 types were the most significantly abundant types within the seven RING finger domains. More RING finger domains were in land plants (embryophytes) than in any other eukaryote or protist species analyzed in this work. With the exception of basal plant *P*. *patens*, there were more RING-H2 type proteins than RING-HC type proteins in embryophytes. This data suggests that the RING-H2 proteins were more expanded than the RING-HC proteins in land plants. In protists and animal species, there were slightly fewer RING-H2 type proteins than RING-HC type proteins. The dominance of the RING-H2 type in land plants and the RING-HC type in animals suggests that a gain and loss of RING proteins occurred within those major phyla.

The other five types of RING finger proteins occur less compared to the RING-H2 and RING-HC types. RING-v and RING-C2 types were expanded in land plants, ranging from 13 to 36 and 5 to 25, respectively. A single RING-v type protein was found in animals and fungi that was absent in protists, *S*. cerevisiae and green algae. At least one RING-C2 type protein was found in all species, except in *T*. *cruzi*. Conversely, RING-S/T and RING-G types were not expanded in embryophytes, ranging from two to six and zero to two respectively; other eukaryotes encoded up to three and one of these types, respectively (Fig 6). This agrees with the fact that the RING-D type was only detected in members of the genus *Arabidopsis*.

### Diversification and specialization of RING finger protein groups

**Fig 7:**
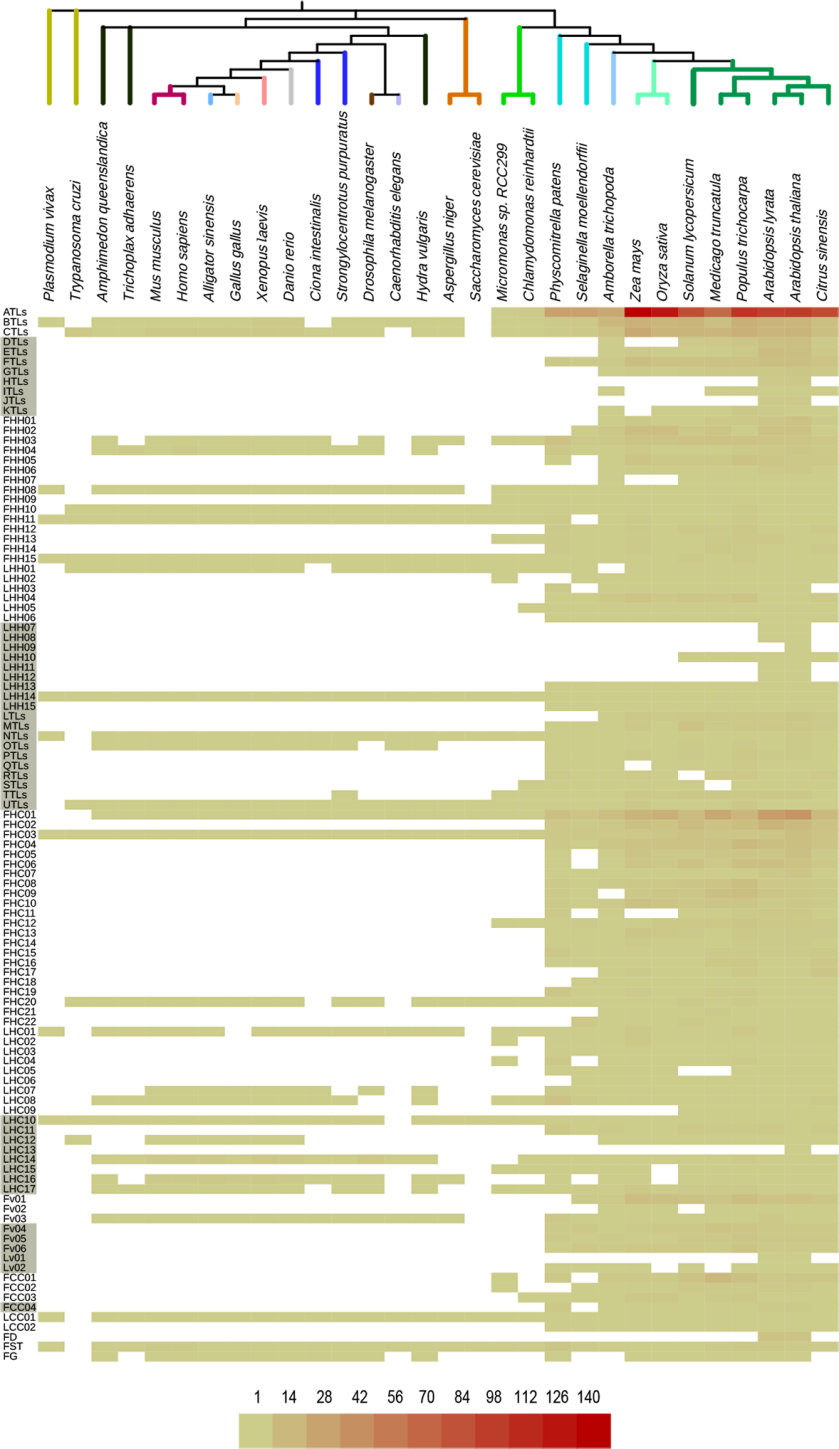
Number of retrieved RING proteins across eukaryotes categorized based on *A*. *thaliana* groups. A heat map shows the number of proteins in each of the 107 RING groups. The fprequency of RING proteins is shown according to a pale green-red scale. The species tree is shown in Fig 6. A detailed distribution of RING identifiers within the groups is displayed in S4 Table.

The group distribution of the 4712 retrieved RING finger protein sequences from 30 species, including *A*. *thaliana*, was established (Fig 7). Out of the 107 RING finger protein groups, 28 included proteins from both plants and animals while some had or lacked protist and/or fungal lineages, suggesting that these groups may be essential for all or most eukaryotes. Notably, two patterns were observed as follows: eleven groups were absent in protists (FHH03, FHH04, OTL, FHC01, LHC07, LHC08, LHC14, LHC16, LHC17, Fv03, and FG), eight groups were absent in fungi (FHH04, OTL, LHC07, LHC08, LHC12, LHC14, LHC17, and FG), and seven groups were common to both patterns (Fig 7).

From the 79 plant specific groups, 14 were included in viridiplantae, five in tracheophytes, 36 in embryophytes (land plants), 12 in angiosperms, and two in eudicots. The prominent number in embryophytes may indicate a major diversification in the transition of plants from water to the land. Noticeable, eight groups are *Arabidopsis* specific (HTLs, JTLs, FD, LHH07, LHH08, LHH11, LHH12, and Lv01), but only two are present in *A*. *thaliana* (LHH09, LHC13) (Fig 7). This fact suggests that specialization of RING-finger proteins is specific to species or lineages.

We also examined the occurrence of the 37 *A*. *thaliana* groups of single RING finger genes denoted as LHC, LHH, LCC, and Lv in other genomes. Ten of these groups were only present in *A*. *thaliana*, but most of them were represented in various genomes in more than a single copy. Twelve groups were represented in more than three plant species and three in animals (S1 Fig). Such a distribution indicates that the copy number for most of the *A*. *thaliana* single RING finger genes is not fixed in the genome.

## Discussion

E3 ubiquitin ligases are abundant in most eukaryotic proteomes, and a variety of E3 ligase families are present in the genomes as multigene families. The expansion of these enzymes is thought to facilitate the active surveillance for targets into the cell by the ubiquitination/26S proteasome pathway. In plants, specifically in *A*. *thaliana*, metal ligands are arranged among the RING domain present in the RING ubiquitin E3 ligase class, and the overall domain architecture was used to previously classify these E3 ligases. Seven types of RING finger domains distributed into 30 different groups were established. We have revised and updated the group organization of the RING finger proteins in *A*. *thaliana*.

The central core sequence of the RING domain was used to seek for RING finger E3 ligase proteins. After excluding proteins lacking boundary metal ligands of the RING domain or annotated as non-RING finger proteins, we put together a highly sequence-related collection of RING proteins than previously reported. The disposition of the four metal ligands encompassing nine to 16 amino acid residues within the central core of the RING domain was highly specific and ample to identify proteins that had at least 75 amino acid residues and a RING domain. This observation was not surprising since the central core of the RING participated in the coordination of two zinc metal ligands that were responsible for the RING domain’s conserved fold [25, 26]. We selected proteins that were not previously annotated. From this central core, the RING finger protein types and most of the inferred RING groups defined in this work branched out. We conceived 3.5 times more RING finger proteins groups than previously defined. We found RING-H2 and RING-HC types to be top ranking in the frequency of RING proteins in a lineage fashion. Similar studies have reported the RING-H2 types to be the most abundant in plants, such as *A*. *thaliana* [10], *B*. *rapa* [12] or *O*. *sativa* [13] as well as the green algae *O*. *tauri* [15]. Meanwhile, the RING-C2, RING-D, RING-S/T, and RING-G type, the RING-HC domains were reported to be the most abundant RING-type in humans [9]. The new grouping was derived, because the domain description for the four previous groups comprising more than 50% of the RING finger proteins was ample and undefined. There were 140 proteins with not identifiable domains in group 1; 20 proteins with coiled-coil domains in group 6; group 24, 107 proteins with one or more transmembrane domains in group 24; and 29 proteins with signal peptides in group 25.

Groups are settled by the presence of known domains or putative motifs shown across the protein sequences outside the RING finger domain. Remarkably, a particular configuration of the RING finger domain is noticeable not only among the seven types of RING finger proteins but also in the subtle variations detected in several groups of the RING-H2 and RING-HC types (Fig 4). One such variant is the proline residue close to the third metal ligand in the RING finger domain which is present in 42% of the groups; functional constraint of this proline was formerly inferred on ATLs and BTLs. Certainly, sequence conservation is detected between RING-H2 and RING-HC types. The tryptophan followed by a hydrophobic residue located at the central region of the domain is particularly conserved in RING-H2. This tryptophan residue is essential since the E3 ligase function is dependent on this residue [27]. On the other hand, length differences among the conserved cysteine and histidine residues are found in RING-HC domains only. It is plausible that RING finger domains in parallel with an array of distinct protein domains go through coordinated changes during evolution to prompt functional innovation and preserve structural features of particular RING finger protein groups. It is tempting to speculate that the RING-G and RING-D types evolved from RING-H2 proteins and that changes were fixed during evolution. This idea is in agreement with the exclusive detection of the RING-D type in plant species belonging to the Brasicaceae family [10, 12]. Our phylogenetic analysis supports this speculation since both types are grouped with RING-H2 sequences (Fig 2). The RING finger protein classification is also supported by the conservation of gene structure within the groups. The incidence and extent of spliceosomal introns is preserved among group members. These groups may primarily consist of intronless genes or a few introns (for example, FHC06, FHH01, FHH07, ATLs, BTLs, DTLs), multiple introns (for example, FHC18, FHC20, FHH03, FHH09), and/or display a 5’UTR intron (for example, FHC06, FHC16, Fv01, BTLs, CTLs, GTLs, PTLs) (Table 3).

Before the general role of RING fingers in ubiquitination was uncovered, the domain was identified in more than 200 proteins across eukaryotes. The RING finger domains are critical for function as an adjustable scaffold that granted functional specificity to proteins that often encode assorted and unrelated domains [28, 29]. Based on the sequence LOGOs domain architecture, exclusive assemblies were generated for most RING finger groups, validating the notion of diverse domain architecture within protein sequences in this group of proteins. It is foreseeable that such architecture exerts a role in functional diversification of RING finger E3 ligases throughout the evolution. The domains and/or conserved motifs may facilitate the recognition of ubiquitination targets in a variety of cell environments, plant tissue, or upon plant confrontation with stress to further ubiquitination. Indeed, transmembrane helices at the amino-terminal are associated with the location of RING E3 ligases in a variety of cell organelles, such as chloroplast (ATL25/NIP2 and OsCTR1) [30, 31], golgi apparatus (OsHCI1) [32], endoplasmic reticulum (SDIR1, Rma1H1, and ATL9) [33–36], nucleus (NLA, BOI, DRIP1/2, and HOS1) [37–40], mitochondria (MITOL/MARCHV) [41], and peroxisome (SPL1) [42]. In addition to the sequence LOGOs mapped to RING finger sequences, only a few LOGOs were common in more than one group. These LOGOs were related in sequence to the previously described GLD motif. GLD and GLD-like motifs were detected in 14 RING-H2 groups and in the RING-D type (Fig 5); the function of these motifs is unknown.

RING finger proteins are diverse across eukaryotic genomes. There are 28 anciently established groups present in both animals and plants that are expected to display essential functions. For example, APC11 (LHH01) controls progression through mitosis and the G1 phase of the cell cycle, while COP1 (LHC08) is involved in plant photomorphogenesis and plays a role in tumorigenesis, gluconeogenesis, and lipid metabolism in animals. Few of these ancient groups show expansion in plant genomes and may have a wide spectrum of functions (for example, BTLs, CTL, BRUTUS (FHH03), RING BETWEEN RING FINGERS (RBR or FHC01 in this work) and SNF2-Helicase (FHC03)). RBR (FHC01) includes the ARIADNE family formerly identified in *Drosophila melanogaster*, *Mus musculus*, and *Homo sapiens* and consists of multiple genes in plants [43–45]. The absence of RING finger groups in animal lineages compared to plants suggests that they emerged after the separation of plants and animals and some of them have expanded to a great extent. Specialization of RING-fingers is readily detected in 10 recent groups that are only present in *A*. *thaliana* or other species of *Arabidopsis* (Fig 7). It is likely that novel types and novel groups of RING finger proteins are present on other species. Indeed, 51 RING finger protein groups were identified in *Brassica rapa* compared to the previous 30 groups defined in *A*. *thaliana* [12].

ATL is the most abundant group of RING finger ligases. There are 20 and 28 members in basal *S*. *moellendorfii* and *P*. *patens*, respectively; 96 and 100 members in grapevines and *A*. *thaliana*, respectively; and 162 members in soybean [16]. ATLs are plant-specific genes that may contain one to three transmembrane helices, the GLD motif, and the distinct RING-H2 domain. Although, only the GLD LOGO is found common to most ATLs (LOGO #4), a diverse set of sequence LOGOs allowed the previous classification of ATLs in nine subfamilies [16]. A predominant function for ATLs is in defense response and abiotic stress. In fact, a co-expression network analysis of grapevine ATL genes suggests a correlation between signaling components involved in immune response and ATLs [19]. Indeed, several ATLs have been associated with the variety of cell membranes, including ATL9, ATL78, ATL55/RING1, ATL25/NIP2, and EL5 [30, 36, 46–48]. The members of this prolific family of RING finger E3 ligases encoding transmembrane helices may interact with specific membrane localized proteins, including signal transduction components which may be controlled by ubiquitination. Moreover, as proposed for ATL55/RING1, they might be involved in signaling from the plasma membrane after coupling with lipid rafts which are the centers for signaling molecule assembly within the plasma membrane.

Functional analysis of most RING finger proteins is in its early stages and few genes have been thoroughly analyzed. Therefore, the biological processes for a variety of RING E3 Ub ligases have been predicted without determination of their targets (for example, GA signaling (BRH1) [49], ABA signaling (RHA2a, XERICO, ATL43) [50–52], pathogen response (RIN2, RIN3, ATL2) [53, 54], and peroxisome biogenesis (PEX10, PEX12) [55, 56]). The substrates for only a small number of them have been identified, including RING E3 ligases with a role in as development plasticity (TEAR1 targets TIE1) [57], ABA signaling (AIP2 targets ABI3, KEG targets ABI5, and AIRP2 targets ATP1/SDIRIP1 [58–60], auxin signaling (SINAT5 targets NAC1) [61], auxin transport and root initiation (XBAT32 targets itself) [62], photomorphogenesis (COP1 targets LAF1, HY5, HYH, cry2, phyA, COL3, HFR1, and AtMYB21; and CIP8 targets HY5 and HYH) [63, 64], carbon/nitrogen nutrient balance response (ATL31 targets 14-3-3 proteins) [65], and iron nutrient balance response (IDF1/ATL14 targets IRT1) [66]. About 40% of the groups established in this work were tagged as undescribed. Similarly, a majority of the genes from described gene families have not yet been annotated with their functions. Our study updated the previous catalog of RING finger proteins plants, based on *A*. *thaliana* proteins. Our study will help with functional characterization and can be used as a reference tool for this fundamental class of regulatory enzymes.

## Materials and methods

### Identification and sequence retrieval of *A*. *thaliana* RING finger proteins

The pattern-matching tool at TAIR was used to search for the signatures shown below the RING sequence (Fig 1A). We selected RING finger proteins by searching the pattern of the central four zinc ligation residues, querying a variable number of residues between the third and fourth zinc ligands and from the first to the third zinc ligands for all types of RING fingers. Variable numbers between the fourth and fifth zinc ligands were inquired for each class, from two to three zinc ligands for RING-H2, RING-HC, RING-D and RING-S/T, from four to five zinc ligands for RING-C2, and seven zinc ligands for RING-v. We then verified the collected proteins for the presence of a RING finger either on the PFAM server or manually with the presence of zinc ligands and confirmed the annotation in the *Arabidopsis* Information Resource (TAIR) database.

### Phylogenetic analysis and sequence alignments

The RING finger sequences were aligned using MUSCLE version 3.8.31. The alignment was used to generate phylogenies using the Find Best DNA/Protein Models tool from MEGA 7.0.20 package with Neighbor-joining (NJ) and Maximum likelihood (ML) models. Analysis was conducted with 1000 bootstrap replicates using Dayhoff (for the 508 RING finger sequences tree), LG (for the 258 RING-H2 sequences tree), or Jones-Taylor-Thornton (for the 196 RING-HC sequences tree) substitution models with 5 gamma categories and the complete deletion option. The tree phylogenies were presented and annotated using the Interactive Tree Of Life (iTOL) v3 at http://itol.embl.de/. Phylogenies for conventional taxonomic classifications were obtained from the National Center of Biotechnology Information (NCBI) taxonomy server http://www.ncbi.nlm.nih.gov/Taxonomy.

### Identification and sequence retrieval of RING finger proteins across eukaryotes

The viridiplantae sequences used in this analysis were retrieved from 12 species: two green algae, two basal embryophytes, one basal angiosperm, two monocots, and five eudicot genomes deposited in the Phytozome 10 database (https://phytozome.jgi. doe.gov/pz/portal.html#). Twelve animal species were surveyed: two basal animal, two basal vertebrates, two invertebrates and six vertebrates. Two protist groups and two of fungi species were also surveyed; sequences were retrieved from the Kyoto Encyclopedia of Genes and Genomes (KEGG) at http://www.genome.jp/kegg/ (species and sequences are found in S1 Table). To identify putative orthologs from each of the selected species, we performed reiterated BLAST searches using entire polypeptide sequences from each of the one hundred *A*. *thaliana* RING finger groups established in this work.

### Generation of sequence LOGOs

To search for conserved motifs in RING finger proteins, we used Multiple EM for Motif Elicitation (MEME) implemented in MEME software (University of Queensland, St. Lucia, Australia) version 4.12.0 (http://meme-suite.org/). The following parameters were used: values of zero or one site per sequence with six and 100 amino acids used as minimum and maximum motif sizes, respectively; and the E-value cutoff was < e-10. We collected non-redundant LOGOs independently from each one of the 36 novel groups of RING finger proteins. No sequence LOGO were generated in ITL, JTL, FHH07, LHH07, LHH08, LHH11, LHH12, LHC13, and Lv01; these groups are only found in *Arabidopsis* or Brasicaseae.

## Supporting information

S1 Fig Occurrence of the thirty-six *A*. *thaliana* groups of “lone” single RING finger genes across eukaryotes. The phylogenetic relationship between thirty genomes was based on the National Center of Biotechnology Information (NCBI) taxonomy server (http://www.ncbi.nlm.nih.gov/Taxonomy) (thirteen viridiplantae, thirteen animals, two fungal, and two protist). The “Lone” RING is listed in Table 1.
(PDF)

S1 Table Retrieved *A*. *thaliana* RING finger proteins. Comparison of list from Stone et al 2005 with this work.
(PDF)

S2 Table Acronyms and codes for RING proteins. (PDF)

S3 Table Catalog of sequence LOGOs. (PDF)

S4 Table Retrieved RING finger proteins across eukaryotes. (PDF)
