# Supplementary material for: Repertoire of plant RING E3 ubiquitin ligases revisited: New groups counting gene families and single genes
Source: PLoS One. 2018 Aug 31;13(8):e0203442. doi: 10.1371/journal.pone.0203442 (PMC6118397; doi:10.1371/journal.pone.0203442)
Supplement: S1 Fig — The phylogenetic relationship between thirty genomes was based on the National Center of Biotechnology Information (NCBI) taxonomy server (http://www.ncbi.nlm.nih.gov/Taxonomy) (thirteen viridiplantae, thirteen animals, two fungal, and two protist). The “Lone” RING is listed in Table 1. (PDF) [file pone.0203442.s001.pdf]

S1 Fig. Occurrence of the thirty-six Arabidopsis groups of "lone" single RING finger genes across eukaryotes.

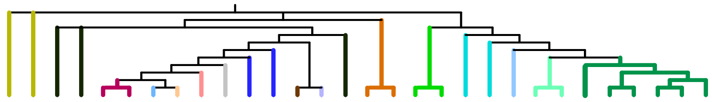

	Plasmodium vivax	*Trypanosoma cruzi*	*Amphimedon queenslandica*	*Trichoplax adhaerens*	*Mus musculus*	*Homo sapiens*	*Alligator sinensis*	*Gallus gallus*	*Xenopus laevis*	*Danio rerio*	*Ciona intestinalis*	*Strongylocentrotus purpuratus*	*Drosophila melanogaster*	*Caenorhabditis elegans*	*Hydra vulgaris*	*Aspergillus niger*	*Saccharomyces cerevisiae*	*Micromonas sp. RCC299*	*Chlamydomonas reinhardtii*	*Physcomitrella patens*	*Selaginella moellendorffii*	*Amborella trichopoda*	*Zea mays*	*Ory a sativa*	*Solanum lycopersicum*	*Medicago truncatula*	*Populus trichocarpa*	*Arabidopsis lyrata*	*Arabidopsis thaliana*	*Citrus sinensis*
LHH01																								2		2		2	1	
LHH02																													1	
LHH03																				3					2	2			1	
LHH04																					2	4	7	4	6	5	7		1	3
LHH05																				2		2							1	
LHH06																							2		2		2		1	
LHH07																													1	
LHH08																													1	
LHH09																													1	
LHH10																											2		1	
LHH11																													1	
LHH12																													1	
LHH13																									2		2		1	
LHH14																										2			1	
LHH15																				2							2		1	
LHC01																				3			5				4		1	
LHC02																				4			4				2		1	
LHC03																								2			2		1	
LHC04																				5						2	3		1	2
LHC05																													1	
LHC06																							2			2	2		1	
LHC07																				3							2		1	
LHC08																				8	3		2		2		2		1	3
LHC09																										2			1	
LHC10		2																											1	
LHC11																				4	2	3	2	2	4	3	3	4	1	3
LHC12																													1	
LHC13																													1	
LHC14					3	3	3	2	5	3			4										2		2	2	3	2	1	
LHC15																													1	
LHC16					3	3	4	3	4	3																			1	
LHC17					2	2	2	2	2	2										2	2		4	2	3	3	3		1	2
Lv01																													1	
Lv02																				2			2				2		1	
LCC01																													1	2
LCC02																							2	2	2		2		1	
