# Supplementary material for: Repertoire of plant RING E3 ubiquitin ligases revisited: New groups counting gene families and single genes
Source: PLoS One. 2018 Aug 31;13(8):e0203442. doi: 10.1371/journal.pone.0203442 (PMC6118397; doi:10.1371/journal.pone.0203442)
Supplement: S1 Table — Comparison of list from Stone et al 2005 with this work. (PDF) [file pone.0203442.s002.pdf]

S1 Table. Retrieved *A. thaliana* RING finger proteins.

RING-HC		RING-H2	
Stone et al, 2005 [10]	This work	Stone et al, 2005 [10]	this work
At1g01350	At1g01350	At1g04360	At1g04360_ATL01
At1g02860	At1g02860	At1g04790	At1g04790
At1g03370	At1g03365		At1g05410
At1g03770	At1g03770	At1g08050	At1g08050
	At1g04020	At1g08190	At1g08190
At1g05120	At1g05120	At1g12470	At1g12470
At1g05880	At1g05880	At1g12760	At1g12760
At1g05890	At1g05890		At1g14180
	At1g06770	At1g14200	At1g14200
At1g10650	At1g10650	At1g15100	At1g15100
At1g11100	At1g11100	At1g17970	At1g17970_CTL15
At1g13195	At1g13195	At1g18910	At1g18910
At1g18470	At1g18470	At1g19680	At1g19680
At1g18660	At1g18660	At1g20823	At1g20823_ATL80
At1g19310	At1g19310	At1g22500	At1g22500_ATL15
At1g21655		At1g22670	At1g22670
	At1g19790	At1g23980	At1g23980_ATL47
At1g22510	At1g22510	At1g24580	At1g24580
At1g24440	At1g24440	At1g26800	At1g26800
At1g30860	At1g30860	At1g27010	
At1g32340	At1g32340	At1g28040	At1g28040_ATL20
At1g32530	At1g32530	At1g32360	At1g32361_ATL93
At1g32740	At1g32740	At1g33480	At1g33480_ATL58
At1g45976	At1g45976	At1g35330	At1g35330_ATL34
	At1g47570	At1g35625	At1g35625
At1g50410	At1g50410	At1g35630	At1g35630
At1g54150	At1g54150		At1g36950_CTL13
At1g55255	At1g55250	At1g45180	At1g45180_CTL5
At1g57800	At1g57800	At1g49200	At1g49200_ATL75
At1g57820	At1g57820	At1g49210	At1g49210_ATL76
At1g59560	At1g59560	At1g49220	At1g49220_ATL10
At1g60610	At1g60610	At1g49230	At1g49230_ATL78
At1g61140	At1g61140	At1g49850	At1g49850
At1g61620	At1g61620	At1g51930	At1g51930_ATL88
At1g62370	At1g62370	At1g53010	At1g53010_ATL19
At1g63900	At1g63900	At1g53190	At1g53190_CTL1
At1g65430	At1g65430	At1g53820	At1g53820_ATL60
At1g66040	At1g66040	At1g55530c	At1g55530_BTL6
At1g66050	At1g66050	At1g57730	At1g57730
At1g66610	At1g66610	At1g60360	At1g60360_BTL17
At1g66620	At1g66620	At1g63170	At1g63170
At1g66630	At1g66630	At1g63840	At1g63840
At1g66650	At1g66650	At1g65040	At1g65040
At1g67800	At1g67800		At1g67856
At1g68820	At1g68820	At1g68070	At1g68070
At1g69330	At1g69330	At1g68180	At1g68180_BTL15
At1g72175	At1g72175	At1g70910	At1g70910
At1g73950	At1g73950	At1g71980	At1g71980
At1g74370	At1g74370	At1g72200	At1g72200_ATL11
At1g74990	At1g74990	At1g72220	At1g72220_ATL54
At1g77770		At1g72310	At1g72310_ATL03
	At1g75520	At1g73760	At1g73760_CTL14
At1g79110	At1g79110	At1g74410	At1g74410_ATL24
At1g79380	At1g79380	At1g74620	At1g74620
At1g79810	At1g79810	At1g74760	At1g74770
At2g14835		At1g75400	At1g75400
At2g16090	At2g16090	At1g76410	At1g76410_ATL08
	At2g18120	At1g80400	At1g80400
At2g19610	At2g19610	At2g01150	At2g01150
At2g21380	At2g21380	At2g01735	At2g01735
	At2g21400	At2g03000	At2g03000
At2g21420	At2g21420	At2g04240	At2g04240
At2g22010	At2g22010	At2g05170	At2g05170
At2g23780	At2g23780		At2g14835
At2g25360	At2g25360	At2g15260	At2g15260
At2g25380	At2g25370	At2g15530	At2g15530_CTL3
At2g26130	At2g26130	At2g15580	At2g15580
At2g26350	At2g26350	At2g17450	At2g17450_ATL44
	At2g27950	At2g17730	At2g17730_ATL25
At2g28840	At2g28840	At2g18650	At2g18650
At2g30580	At2g30580		At2g18650_ATL49
At2g31510	At2g31510	At2g18670	At2g18670_ATL56
At2g31760	At2g31760	At2g20030	At2g20030_ATL12
At2g31770	At2g31770	At2g20650	At2g20650
At2g31780	At2g31780	At2g21500	At2g21500
At2g32950	At2g32950	At2g22680	At2g22680
At2g34920	At2g34920	At2g24480	At2g24480
At2g35330	At2g35330	At2g25410	At2g25410_ATL22
At2g37150	CTL10	At2g26000	At2g26000
	At2g38185	At2g27940	At2g27940_ATL57
At2g38190	At2g38195	At2g28920	At2g28920_ATL84
	At2g38220		At2g34000_ATL99
At2g38920	At2g38920	At2g34990	At2g34990_ATL38
At2g39100	At2g39100	At2g35000	At2g35000_ATL09
	At2g39810	At2g35420	At2g35420_ATL28
At2g40770	At2g40770	At2g35910	At2g35910_ATL70
At2g41980	At2g41980		At2g37150.3_CTL10
At2g42030	At2g42030	At2g37580	At2g37580_ATL33
At2g42160		At2g38970	At2g38970
At2g44410	At2g44410	At2g39720	At2g39720_BTL11
At2g44580		At2g40830	At2g40830_BTL1
At2g44950	At2g44950		At2g42160
At2g46495	ATL21	At2g42350	At2g42350_ATL40
	At2g45135	At2g42360	At2g42360_ATL41
At2g47090	At2g47090	At2g44330	At2g44330_BTL14
At3g01650	At3g01650		At2g44578_ATL89
At3g05250	At3g05250		At2g44581_ATL90
At3g05670		At2g46160	At2g46160_ATL67
At3g06140	At3g06140		At2g46493_ATL95
At3g07120	At3g07120		At2g46494_ATL94
At3g07200	At3g07200		At2g46495_ATL21
At3g08505	At3g08505	At2g47560	At2g47560_ATL64
At3g09770	At3g09770	At2g47700	At2g47700
At3g12920	At3g12920	At3g02290	At3g02290
At3g14250a	At3g14250	At3g02340	At3g02340
At3g16600	At3g16600	At3g03550	At3g03550_ATL51
At3g20010	At3g20010	At3g05200	At3g05200_ATL06
At3g23060	At3g23060	At3g05545	At3g05545
At3g23280	At3g23280	At3g05870	At3g05870
At3g24800	At3g24800	At3g10810	
At3g25030	At3g25030		At3g10815_BTL3
At3g26730	At3g26730	At3g10910	At3g10910_ATL72
At3g27330	At3g27330	At3g11110	At3g11110_ATL66
At3g27710	At3g27710	At3g13430	At3g13430_BTL7
	At3g27720	At3g14320	At3g14320_ATL61
At3g28880	At3g28880	At3g14970	At3g14970
At3g29270	At3g29270		At3g14980
At3g30733		At3g15070	At3g15070_CTL2
At3g43180	At3g43180	At3g15740	At3g15740
At3g43750	At3g43750	At3g16090	At3g16090
	At3g45460	At3g16720	At3g16720_ATL02
At3g45470	At3g45470	At3g18290	At3g18290
At3g45480	At3g45480	At3g18773	At3g18773_ATL77
At3g45510	At3g45510		At3g18777_ATL81
At3g45540	At3g45540	At3g18930	At3g18930_ATL65
At3g45555	At3g45555		At3g19140_ATL62
At3g45560	At3g45560	At3g19910	At3g19910_CTL18
At3g45570	At3g45570	At3g19950	At3g19950_BTL8
At3g45580	At3g45580		At3g20395_ATL92
At3g47160	At3g47160	At3g28620	At3g28620
	At3g51060	At3g30460	At3g30460
At3g53410	At3g53410	At3g42830	At3g42830
At3g53690	At3g53690	At3g43430	At3g43430
At3g54360	At3g54360	At3g46620	At3g46620_BTL10
	At3g54430	At3g47180	At3g47180_CTL16
At3g54460	At3g54460	At3g47990	At3g47990
At3g58030	At3g58030	At3g48030	At3g48030_ATL48

RING-v		RING-S/T		RING-D		RING-G		RING-C2	
Stone et al, 2005 [10]	This work	Stone et al, 2005 [10]	This work	Stone et al, 2005 [10]	This work	Stone et al, 2005 [10]	This work	Stone et al, 2005 [10]	This work
At1g02610	At1g02610	At1g36950	CTL13	At1g18760	At1g18760	At5g03450	At5g03450		At1g05055
At1g11020	At1g11020	At2g22690	At2g22690	At1g18770	At1g18770			At1g09060	At1g09060
At1g14260	At1g14260	At3g19140	ATL62	At1g18780	At1g18780			At1g11950	At1g11950
At1g50440	At1g50440	At4g37880	At4g37880	At1g19030					At1g20110
At2g01275	At2g01275		At5g09630	At1g21960	At1g21960				At1g29800
At2g02960	At2g02960			At2g11348	not found			At1g62310	At1g62310
At2g22120	At2g22120			At2g29840	At2g29840			At1g74870	At1g74870
At2g34200	At2g34200			At3g13228	At3g13228			At2g28530	At2g28540
At2g37950	At2g37950			At5g53910	At5g53910			At3g07610	At3g07610
At2g45530	At2g45530			At2g34000	ATL99				At3g43230
At3g06330	At3g06330							At3g45630	At3g45630
At3g09760	At3g09760							At3g48070	At3g48070
At3g47550	At3g47550							At4g00990	At4g00990
At4g02075	At4g02075								At4g21430
	At4g32670								At5g60170
At4g34100	At4g34100								At5g62910
At5g01070	At5g01070								
At5g03180	At5g03180								
At5g05830	At5g05830								
At5g08750	At5g08750								
At5g18760	At5g18760								
At5g38070	At5g38070								
At5g59000	At5g59000								
	At5g60580								
	At5g62460								
	At5g63780								

At3g58040	At3g58040		At3g51325
At3g61790	At3g61790	At3g54780	At3g54780
At3g62240	At3g62240	At3g55530	At3g55530
At4g00070	CTL12	At3g56575	At3g56580_BTL2
At4g01020	At4g01020	At3g58720	At3g58720
At4g01023	At4g01023	At3g60080	At3g60080_BTL13
At4g03000	At4g03000	At3g60220	At3g60220_ATL04
At4g03510	At4g03510	At3g60300	At3g60300
At4g03965	At4g03965		At3g60966_ATL91
At4g08460		At3g61180	At3g61180
At4g08590	At4g08590	At3g61460	At3g61460
At4g10940		At3g61550	At3g61550_ATL68
At4g13100	At4g13100	At3g62690	At3g62690_ATL05
At4g14365	At4g14365	At3g62970	At3g62970
At4g17680	At4g17680	At3g63530	At3g63530_CTL19_BB
At4g19670	At4g19670		At4g00070_CTL12
At4g19700	At4g19700	At4g00305	At4g00305
At4g21070	At4g21070	At4g00335	At4g00335
	At4g21110	At4g01270	At4g01270
At4g22250	At4g22250	At4g05350	At4g05350
	At4g24204	At4g09100	At4g09100_ATL39
At4g27470	At4g27470	At4g09110	At4g09110_ATL35
At4g27880	At4g27880	At4g09120	At4g09120_ATL36
At4g28270	At4g28270	At4g09130	At4g09130_ATL37
At4g33940	At4g33940	At4g09560	At4g09560
At4g34370	At4g34370	At4g10150	At4g10150_ATL07
At4g35070	At4g35070	At4g10160	At4g10160_ATL59
	At4g36260	At4g11360	At4g11360
At4g39050	At4g39050	At4g11370	At4g11370
At5g01160	At5g01160	At4g11680	At4g11680
At5g01450	At5g01450	At4g12140	At4g12140
At5g01520	At5g01520	At4g12150	At4g12150
At5g01960	At5g01960	At4g12190	At4g12190
At5g03200	At5g03200	At4g12210	At4g12210
	At5g04460	At4g13490	At4g13490
At5g05130	At5g05130	At4g14220	At4g14220
At5g06420	At5g06420	At4g15975	At4g15975_ATL17
At5g07270	At5g07270	At4g17245	At4g17245_ATL18
At5g07640	At5g07640	At4g17910	At4g17905_ATL53
	At5g08730	At4g17920	At4g17920_ATL29
At5g10370	At5g10370	At4g18110	At4g18110
At5g12310	At5g12310	At4g23450	At4g23450
At5g13530	At5g12330		At4g24015_ATL82
	At5g13530	At4g25230	At4g25230
At5g14420	At5g14420	At4g26400	At4g26400_BTL5
At5g19080	At5g19080	At4g26580	At4g26580
At5g19430	At5g19430	At4g28370	At4g28370
At5g22750	At5g22750	At4g28890	At4g28890_ATL42
At5g23110	At5g23110	At4g30370	At4g30370_ATL14
	At5g33210	At4g30400	At4g30400_ATL13
At5g37560	At5g37560	At4g31450	At4g31450_CTL9
At5g37870	At5g37870	At4g32600	At4g32600
At5g37890	At5g37890	At4g33565	At4g33565_ATL83
At5g37910	At5g37910	At4g34040	At4g34040_CTL4
At5g37930	At5g37930	At4g35480	At4g35480_ATL45
At5g39550	At5g39550	At4g35840	At4g35840_ATL26
At5g43530	At5g43530	At4g37890	At4g37890
At5g44280	At5g44280	At4g38140	At4g38140_ATL100
At5g44690		At4g39140	At4g39140
At5g45100	At5g45100	At4g40070	At4g40070_ATL32
At5g47050	At5g47050	At5g01880	At5g01880_ATL74
At5g48655	At5g48655	At5g01980	At5g01980_BTL16
At5g53360	At5g53360	At5g02750	At5g02750
At5g57740	At5g57740	At5g05280	At5g05280_ATL73
At5g58787	At5g58787	At5g05530	
	At5g62800	At5g05810	At5g05810_ATL43
	At5g63730	At5g05910	At5g05910_ATL98
	At5g63740	At5g06490	At5g06490_ATL71
	At5g63750	At5g07040	At5g07040_ATL69
	At5g63760	At5g07225	At5g07225
	At5g63970	At5g08139	At5g08139
	At5g66350	At5g10380	At5g10380_ATL55
		At5g10650	At5g10650_CTL8
		At5g15790	At5g15790
		At5g15820	At5g15820
		At5g17600	At5g17600_ATL52
		At5g18260	At5g18260
		At5g18650	At5g20570
		At5g20570	
		At5g20885	At5g20885
		At5g20910	At5g20910
		At5g22000	At5g22000
		At5g22920	At5g22920
		At5g24870	At5g24870_CTL7
		At5g25560	At5g25560
		At5g27420	At5g27420_ATL31
			At5g36001_ATL97
		At5g37200	At5g37200
			At5g37220
		At5g37230	At5g37230
		At5g37250	At5g37250
		At5g37270	At5g37270
		At5g37280	At5g37280
		At5g38895	At5g38895
		At5g40250	At5g40250_ATL46
		At5g41350	At5g41350
		At5g41400	At5g41400
		At5g41430	At5g41430_ATL85
		At5g41440	At5g41440_ATL86
		At5g41450	At5g41450_ATL87
		At5g42200	At5g42200_ATL23
		At5g42940	At5g42940_CTL6
		At5g43200	At5g43200
		At5g43420	At5g43420_ATL16
		At5g45290	At5g45290
		At5g46650	At5g46650_ATL30
		At5g47610	At5g47610_ATL79
		At5g49665	At5g49665
		At5g51450	At5g51450
			At5g52140_CTL17
			At5g53110_ATL96
		At5g54990	At5g54990
		At5g55970	At5g55970
		At5g56340	At5g56340_BTL4
		At5g57750	At5g57750_ATL50
		At5g57820	At5g57820
		At5g58580	At5g58580_ATL63
			At5g59550_BTL9
			At5g60710
			At5g60820
			At5g64920_BTL12
			At5g65683
			At5g66070_ATL27
			At5g66160
			At5g67120_CTL11

obsolete or bad annotation, and/or change numeral

cysteine-rich domains, not selected as RING finger domain

formerly listed as RING-HC type finger, matches RING-H2 type finger
