# Supplementary material for: Repertoire of plant RING E3 ubiquitin ligases revisited: New groups counting gene families and single genes
Source: PLoS One. 2018 Aug 31;13(8):e0203442. doi: 10.1371/journal.pone.0203442 (PMC6118397; doi:10.1371/journal.pone.0203442)
Supplement: S2 Table — (PDF) [file pone.0203442.s003.pdf]

S2 Table. Acronyms and codes for RING proteins

Acronym/code	RING-type	Status	Connotation
ATL to CTL	RING-H2	Described	A-Type RING Ligase family to C-Type Ligase family
DTL to KTL	RING-H2	Undescribed	D-Type RING Ligase family to K-Type Ligase family
LTL to UTL	RING-HC	Undescribed	L-Type RING Ligase family to V-Type Ligase family
FHC01 to FHC22	RING-HC	Described	Family RING-HC 01 to Family RING-HC 22
FHH01 to FHH15	RING-H2	Described	Family RING-H2 01 to Family RING-H2 15
Fv01 to Fv03	RING-v	Described	Family RING-v
Fv04 to Fv06	RING-v	Undescribed	Family RING-v
FCC01 to FCC03	RING-C2	Described	Family RING-C2
FCC04	RING-C2	Undescribed	Family RING-C2
FD	RING-D	Described	Family RING-D
FS/T	RING-S/T	Described	Family RING-S/T
FG	RING-G	Described	Family RING-G
LHH01 to LHH06	RING-H2	Described	Lone RING-H2
LHH07 to LHH15	RING-H2	Undescribed	Lone RING-H2
LHC01 to LHC09	RING-HC	Described	Lone RING-HC
LHC10 to LHC17	RING-HC	Undescribed	Lone RING-HC
Lv01 to Lv02	RING-v	Undescribed	Lone RING-v
LCC01 to LCC02	RING-C2	Described	Lone RING-C2
