# Supplementary material for: Repertoire of plant RING E3 ubiquitin ligases revisited: New groups counting gene families and single genes
Source: PLoS One. 2018 Aug 31;13(8):e0203442. doi: 10.1371/journal.pone.0203442 (PMC6118397; doi:10.1371/journal.pone.0203442)
Supplement: S3 Table — (PDF) [file pone.0203442.s004.pdf]

S3 Table.

Catalog of sequence LOGOs generated from RING finger protein types.

RING-H2	LOGO#	LOGO sequence
ATL	(4)	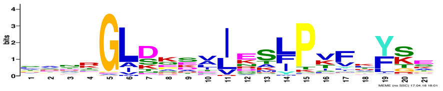
BTL	(28)	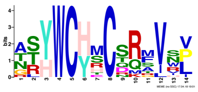
	(38)	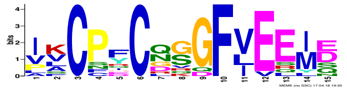
	(11)	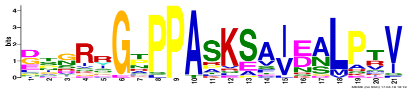
CTL	(5)	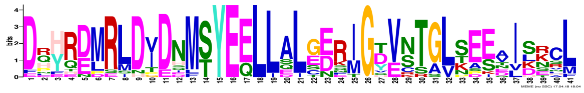
DTL	(11)	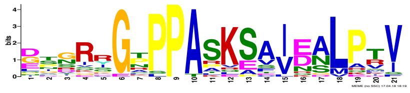
ETL	(13)	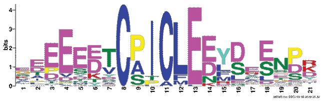
FTL	(8)	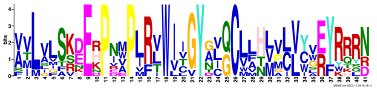
	(10)	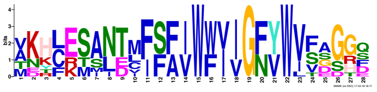
	(4)	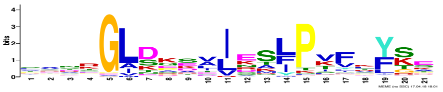
GTL	(62)	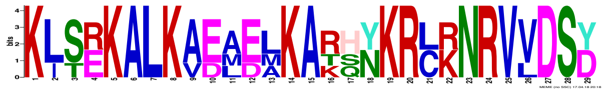
HTL	(23)	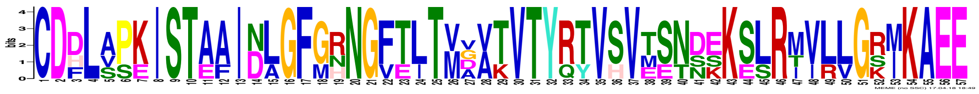
KTL	(189)	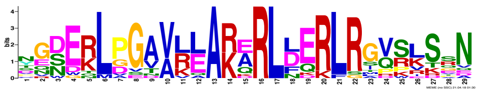
FHH01	(65)	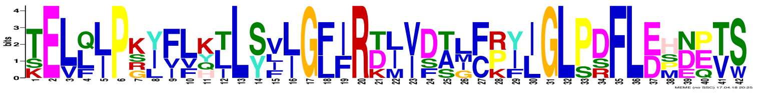

FHH02 (12)

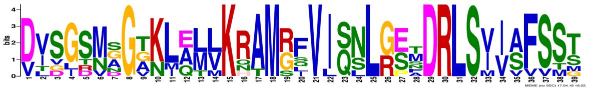

(36)

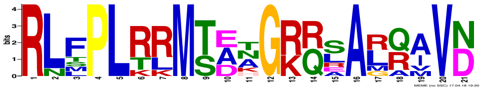

(14)

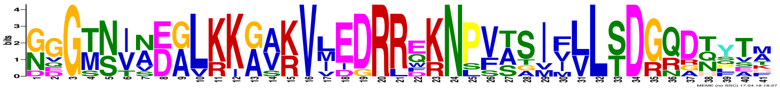

FHH03 (95)

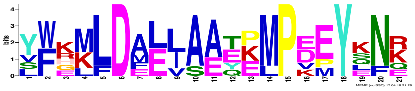

(19)

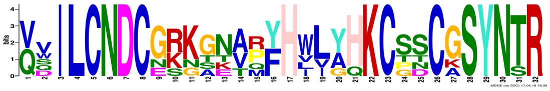

FHH04 (97)

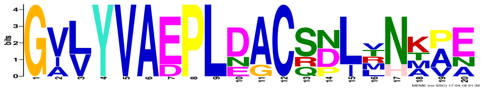

(15)

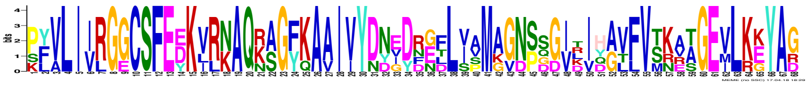

(25)

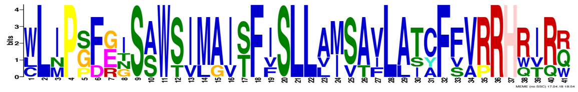

FHH05 (84)

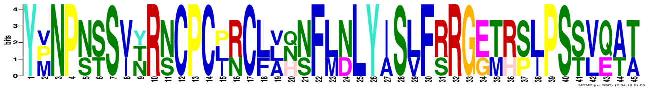

FHH06 (174)

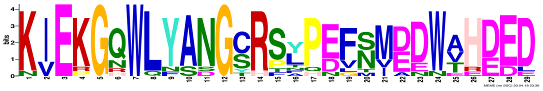

(160)

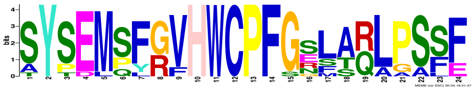

(191)

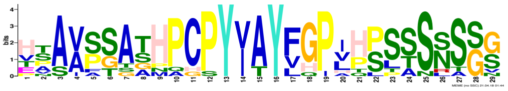

FHH08 (33)

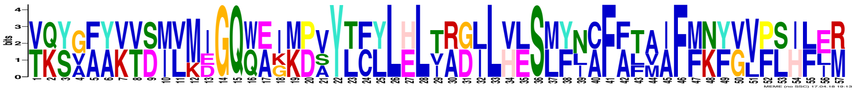

(39)

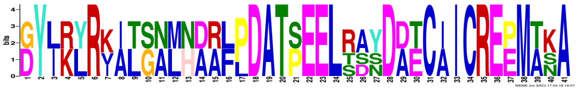

FHH09 (55)

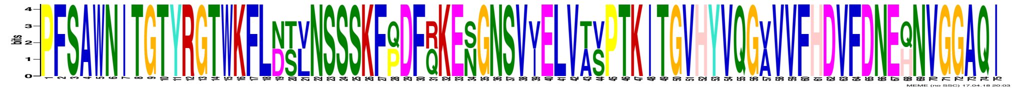

(80)

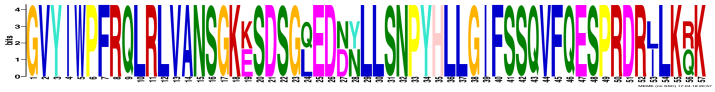

FHH10 (56)

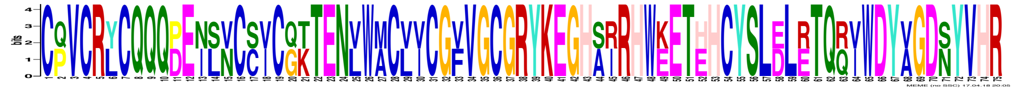

FHH11 (103)

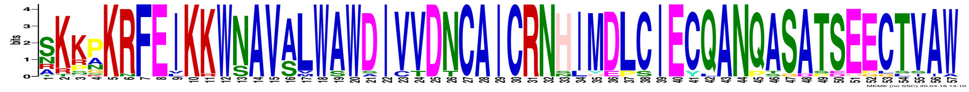

(195)

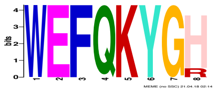

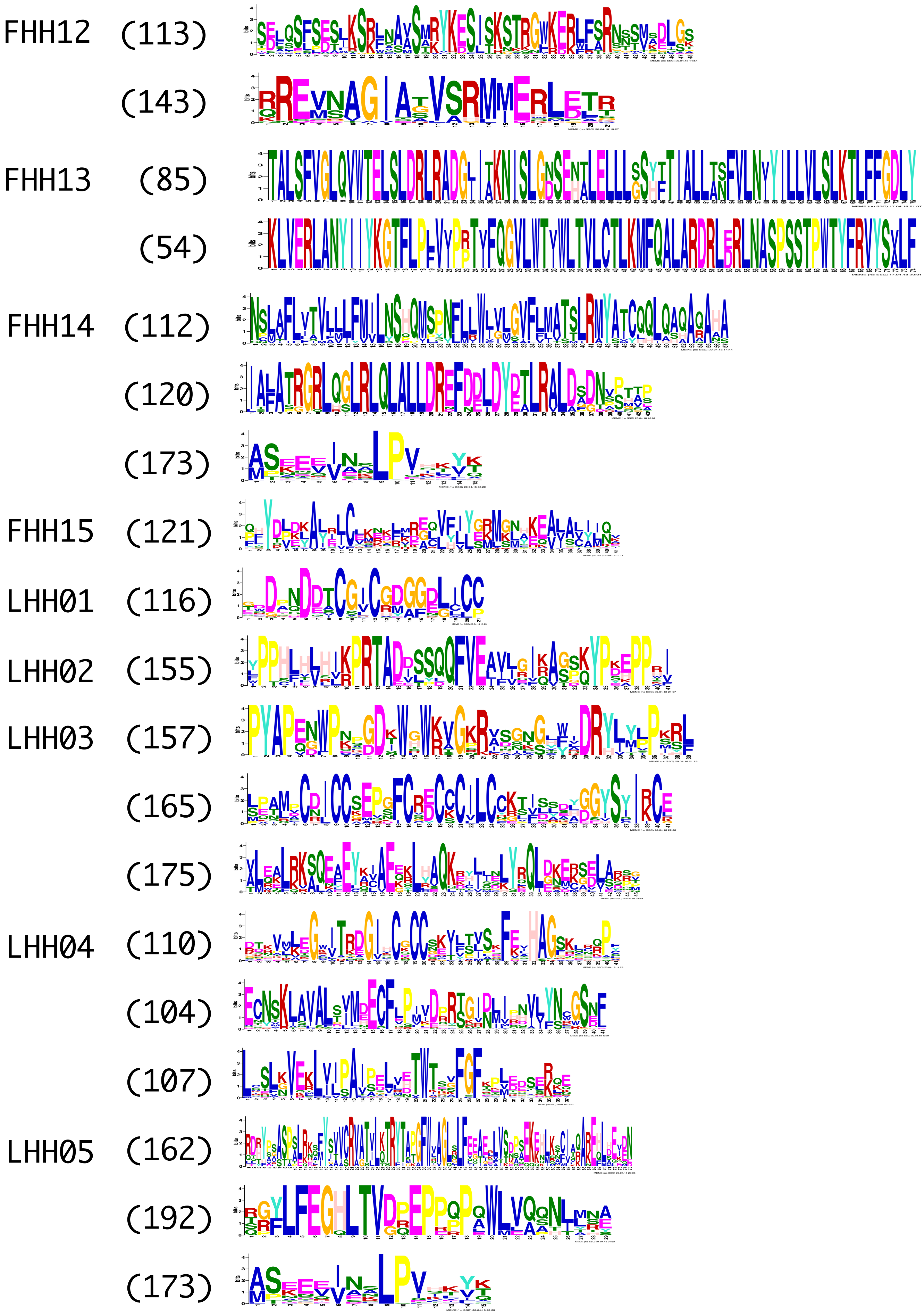
FHH12 (113)
(143)
FHH13 (85)
(54)
FHH14 (112)
(120)
(173)
FHH15 (121)
LHH01 (116)
LHH02 (155)
LHH03 (157)
(165)
(175)
LHH04 (110)
(104)
(107)
LHH05 (162)
(192)
(173)

LHH06 (154)

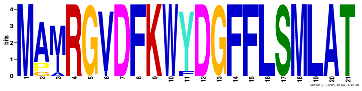

(105)

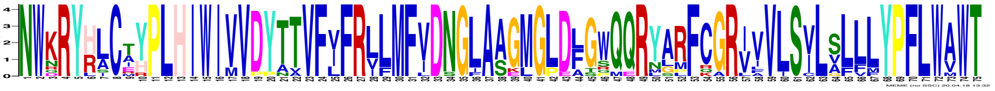

(117)

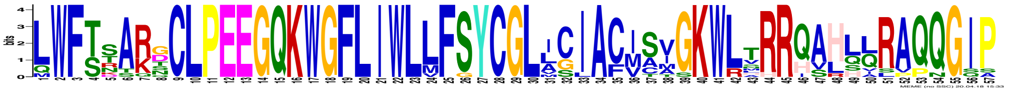

LHH09 (173)

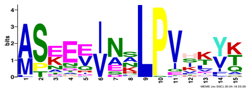

LHH10 (168)

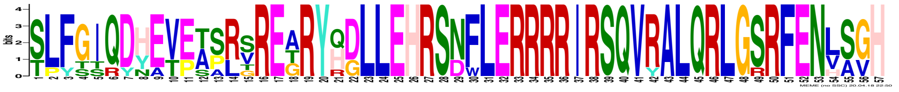

(190)

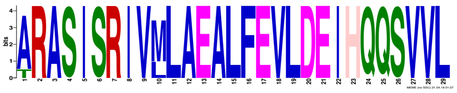

(173)

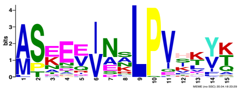

LHH13 (100)

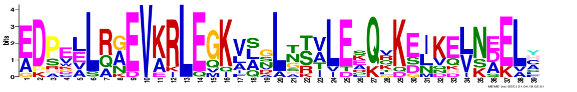

(148)

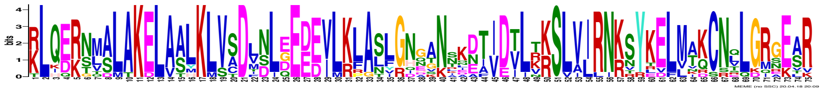

LHH14 (135)

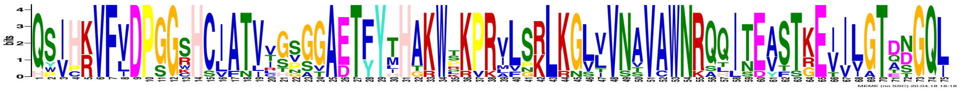

(193)

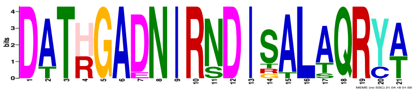

(126)

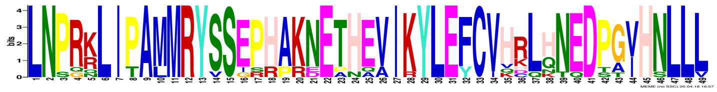

LHH15 (124)

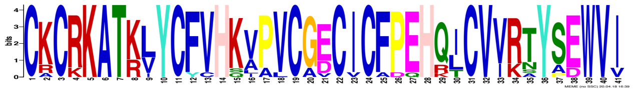

(170)

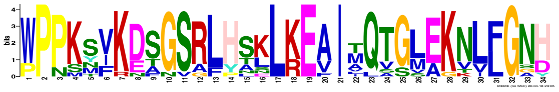

(164)

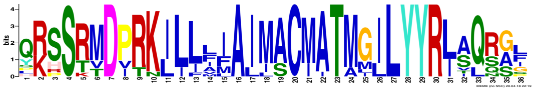

RING-HC	LOGO#	LOGO sequence
LTL	(114)	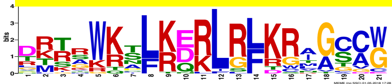
MTL	(61)	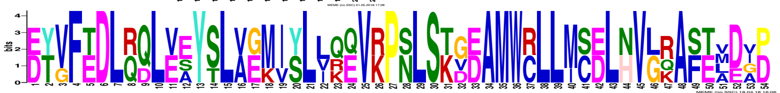
	(64)	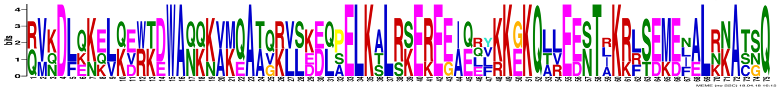
	(63)	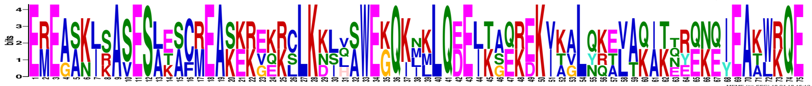
NTL	(66)	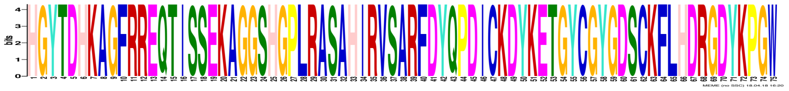
OTL	(160)	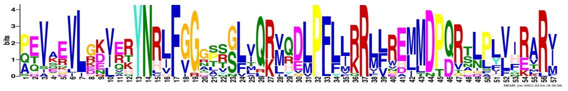
PTL	(118)	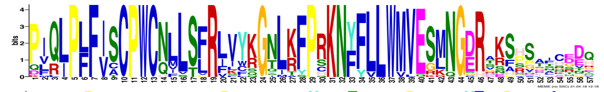
	(106)	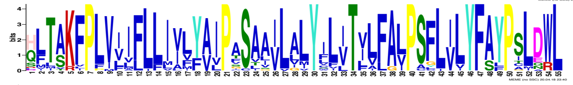
QTL	(145)	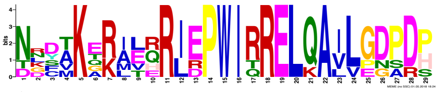
RTL	(219)	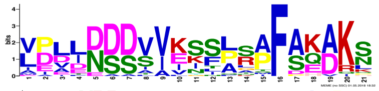
STL	(109)	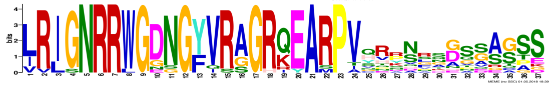
	(217)	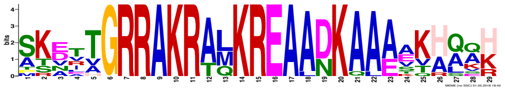
TTL	(70)	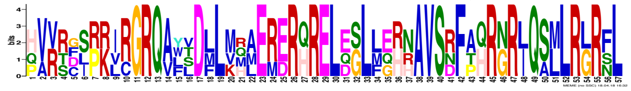
UTL	(75)	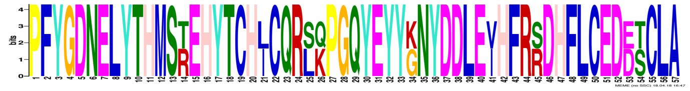
FHC01	(8)	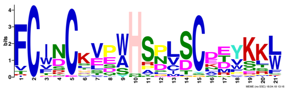
FHC02	(21)	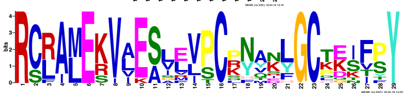
FHC03	(6)	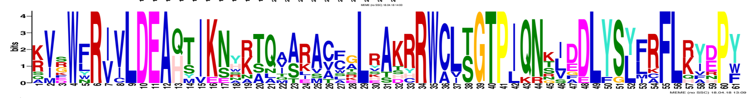
	(37)	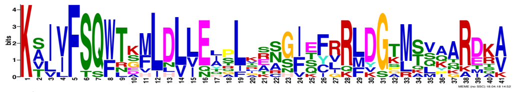
	(3)	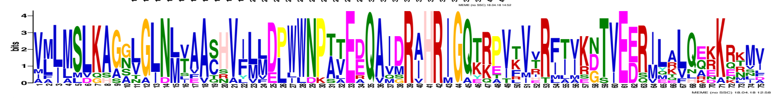
FHC04	(71)	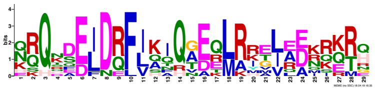
	(15)	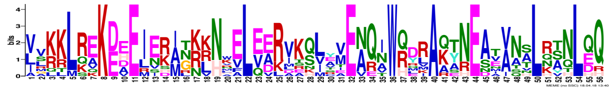
FHC05	(99)	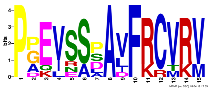
	(23)	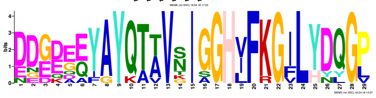

FHC06 (132)

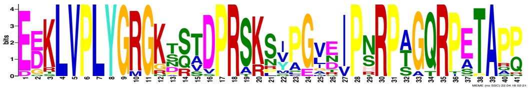

(174)

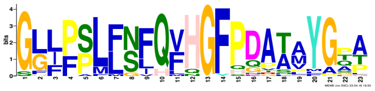

FHC07 (10)

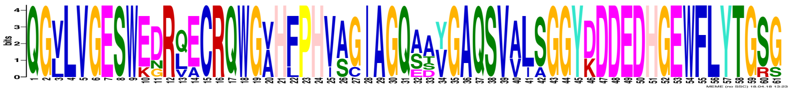

(28)

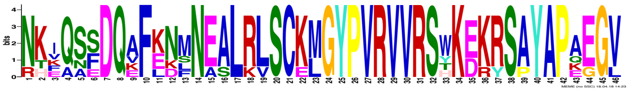

FHC08 (60)

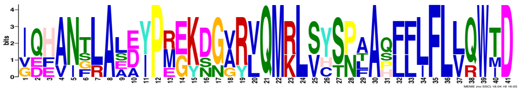

(49)

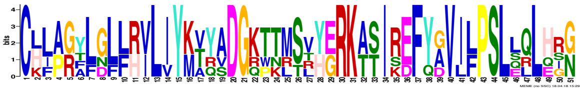

FHC09 (17)

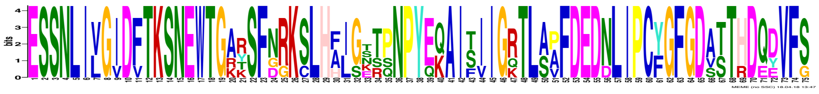

(32)

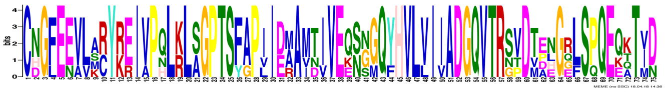

(30)

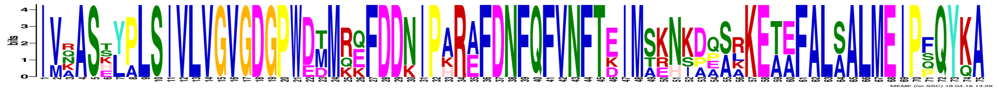

FHC10 (62)

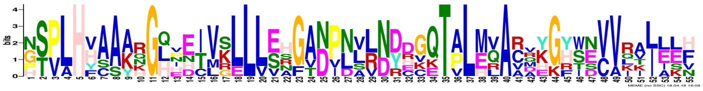

FHC11 (46)

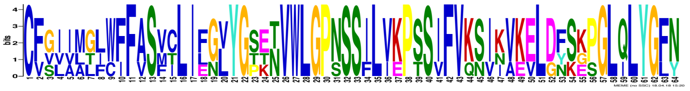

(50)

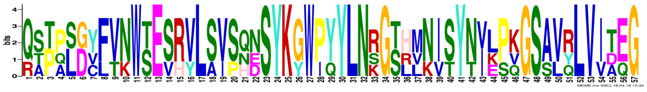

(57)

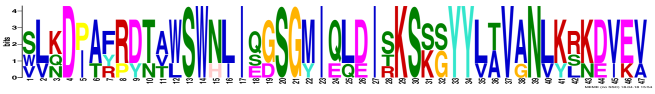

FHC12 (97)

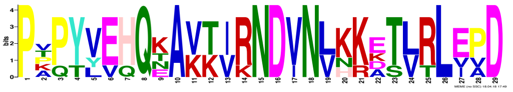

(36)

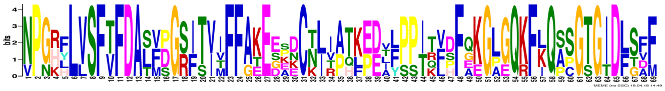

FHC13 (173)

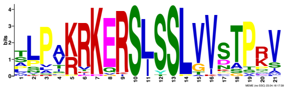

(111)

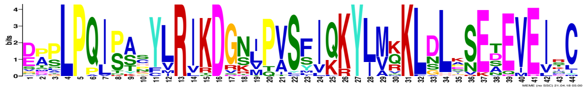

(188)

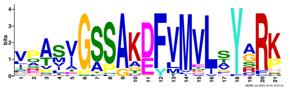

FHC14 (65)

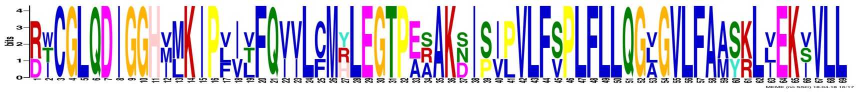

(83)

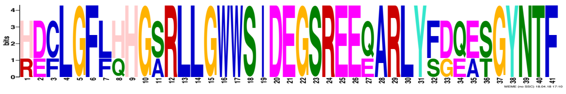

(84)

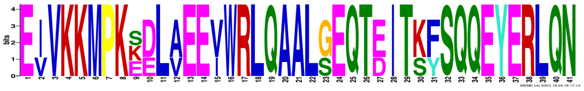

FHC15 (124)

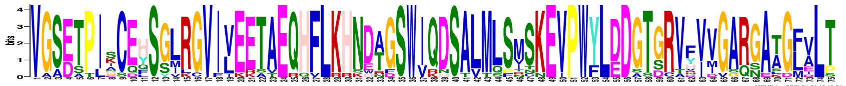

(122)

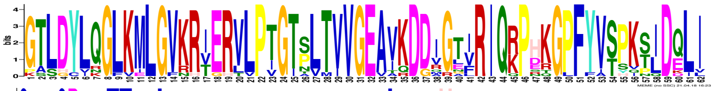

FHC16 (144)

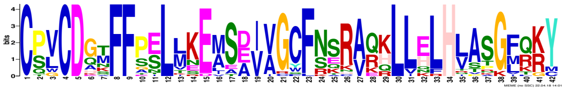

(113)

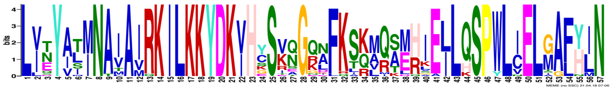

(153)

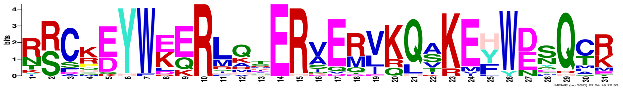

FHC17 (117)

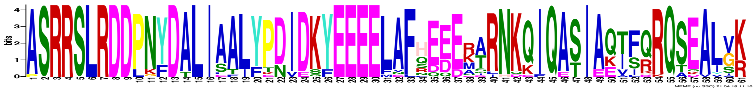

(147)

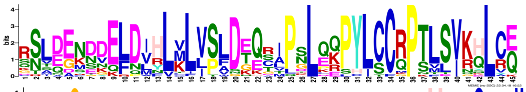

FHC18 (178)

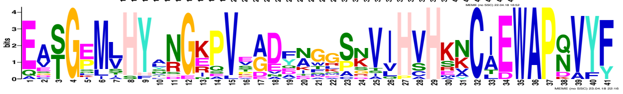

(128)

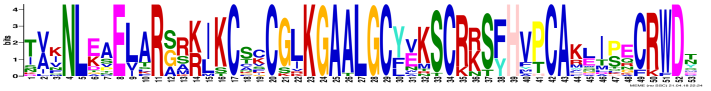

(119)

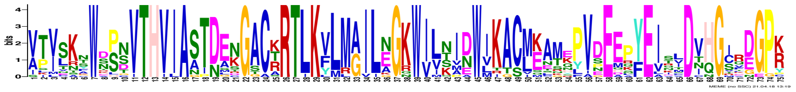

FHC19 (123)

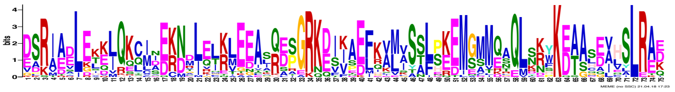

(133)

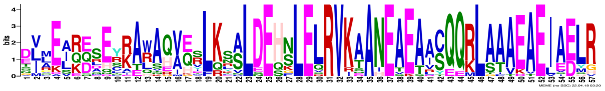

(116)

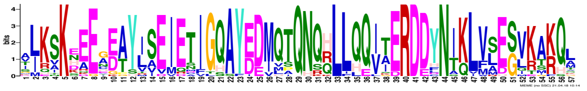

FHC20 (104)

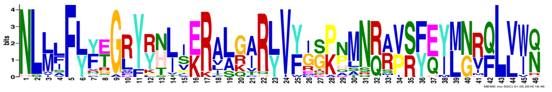

FHC21 (78)

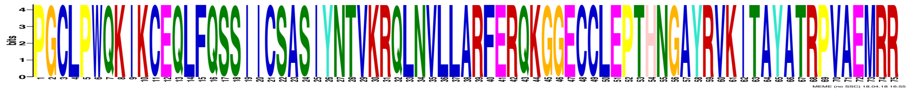

(80)

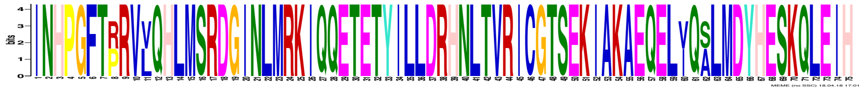

(93)

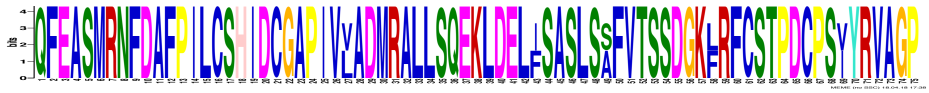

FHC22 (81)

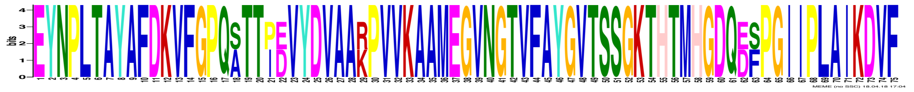

(89)

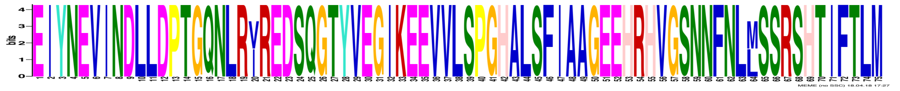

(87)

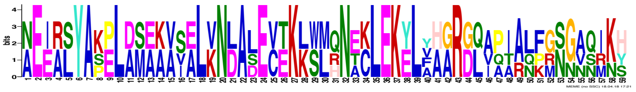

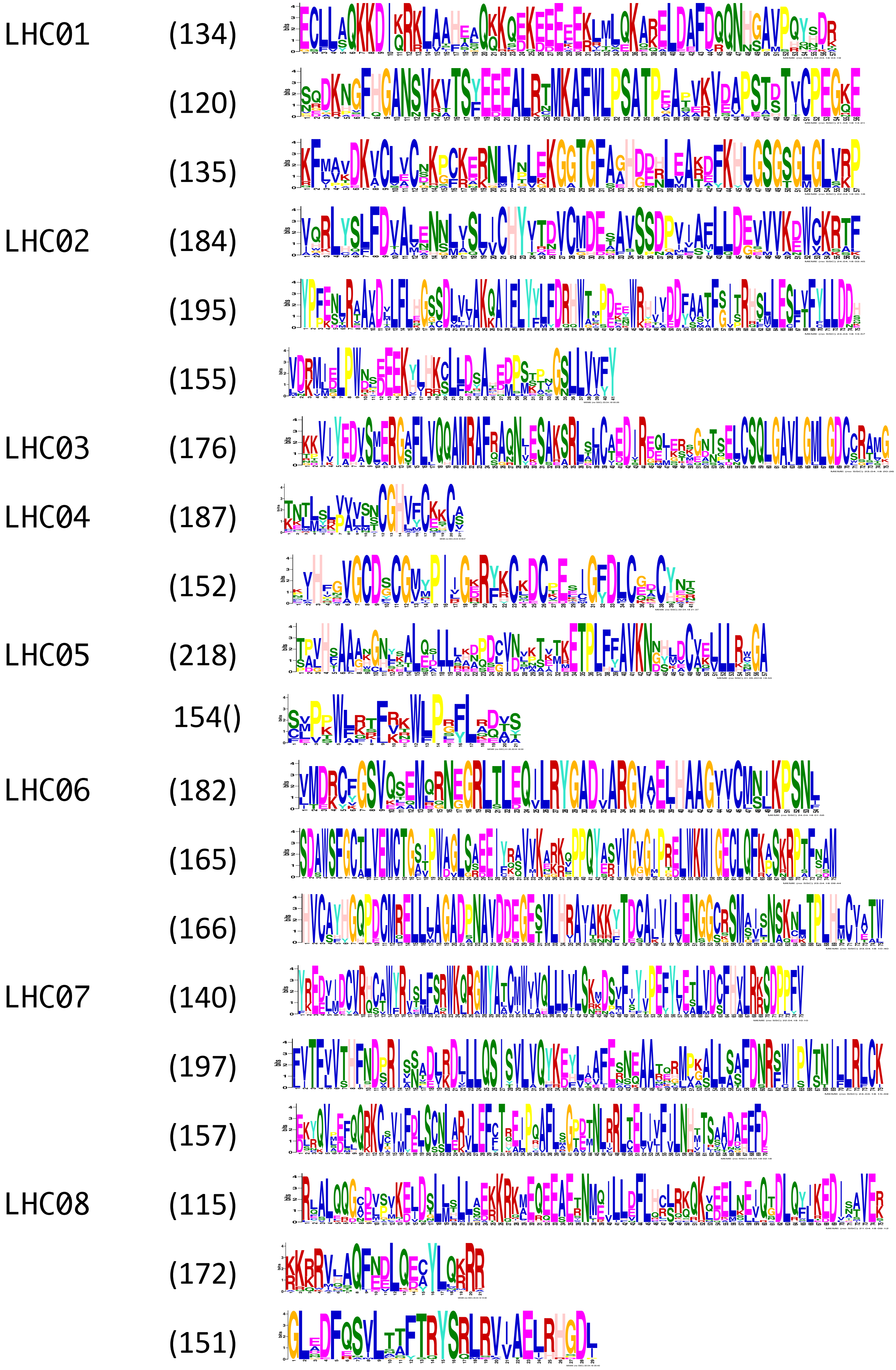

LHC01 (134)
(120)
(135)
LHC02 (184)
(195)
(155)
LHC03 (176)
LHC04 (187)
(152)
LHC05 (218)
154()
LHC06 (182)
(165)
(166)
LHC07 (140)
(197)
(157)
LHC08 (115)
(172)
(151)

LHC09 (116)

(134)

(127)

LHC10 (175)

LHC11 (108)

(137)

(112)

LHC12 (120)

LHC14 (131)

(136)

(125)

LHC15 (186)

(164)

(185)

LHC16 (183)

LHC17 (105)

(170)

(109)

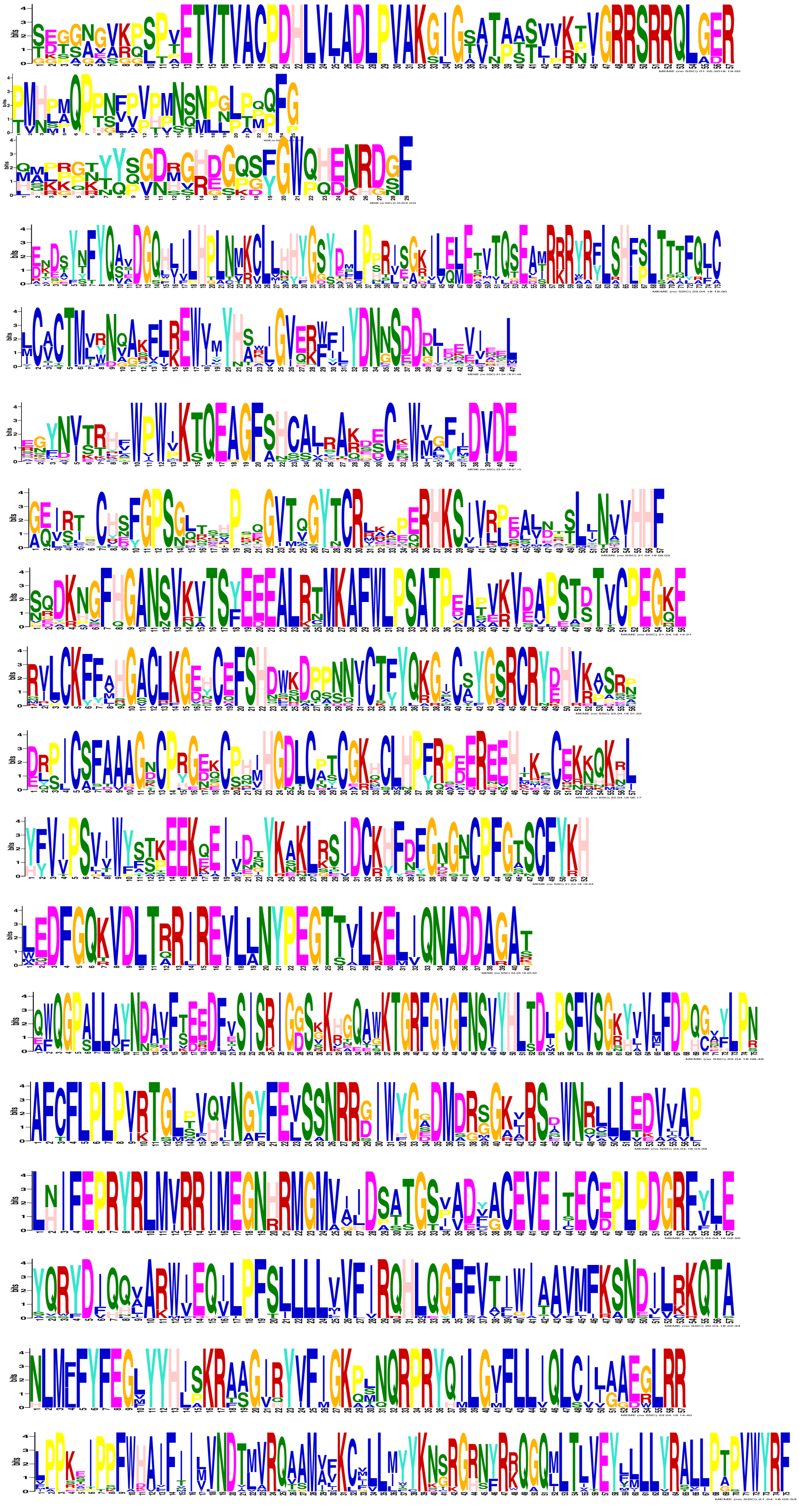

RING-C2	LOGO#	LOGO sequence
FCC01	(4)	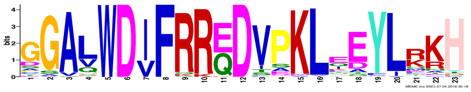
	(1)	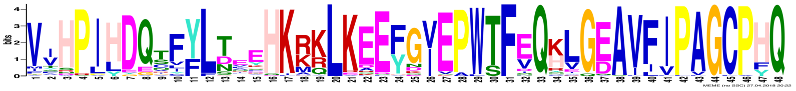
	(3)	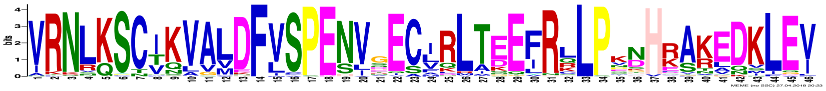
FCC02	(23)	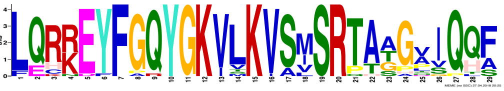
	(5)	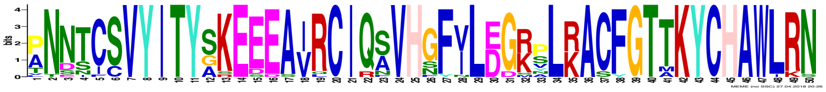
	(27)	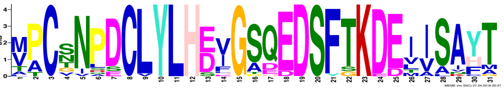
FCC03	(10)	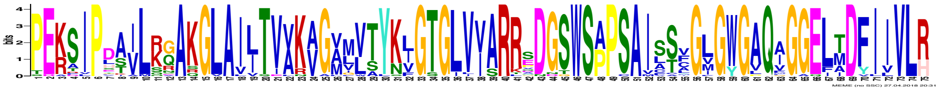
	(13)	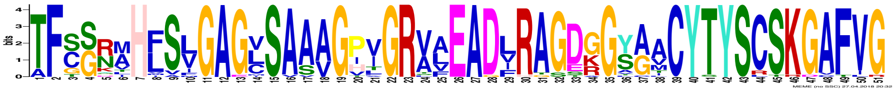
	(41)	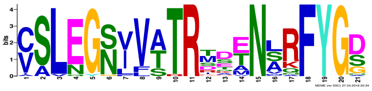
FCC04	(47)	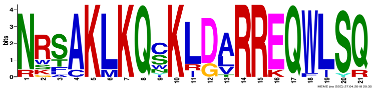
	(93)	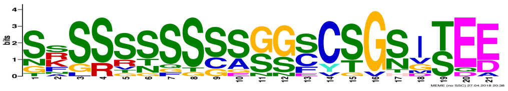
	(46)	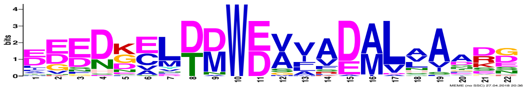
LCC01	(26)	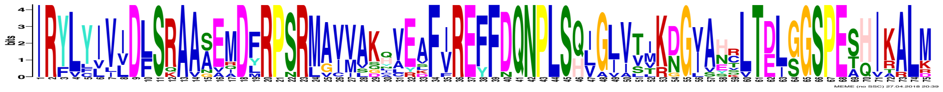
	(38)	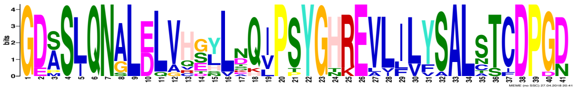
	(19)	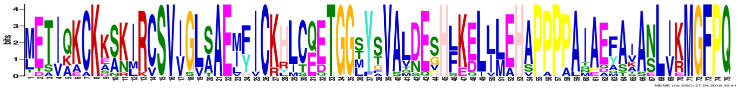
LCC02	(66)	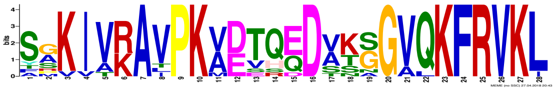
	(35)	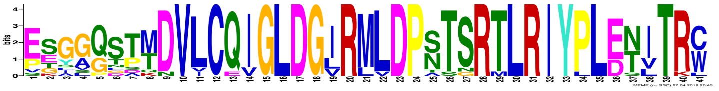
	(25)	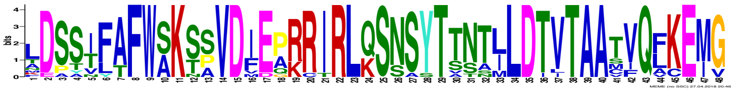

RING-Fv	LOGO#	LOGO sequence
Fv01	(5)	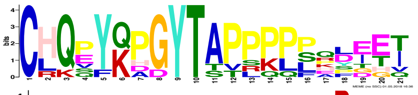
	(4)	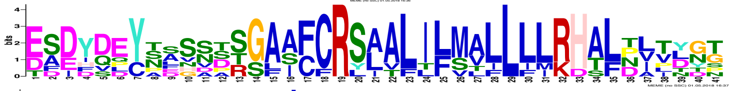
	(6)	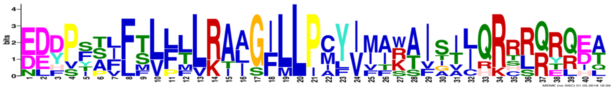
Fv02	(15)	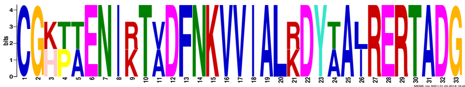
	(16)	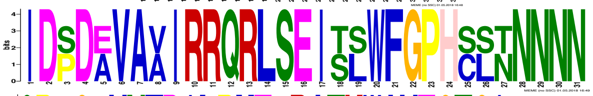
	(13)	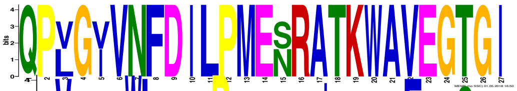
Fv03	(26)	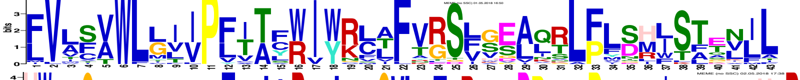
	(2)	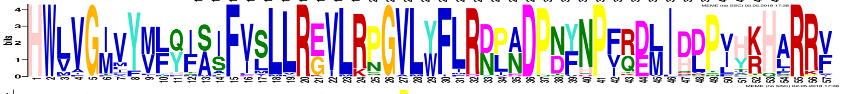
	(27)	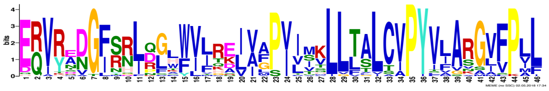
Fv04	(11)	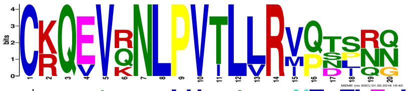
Fv04	(3)	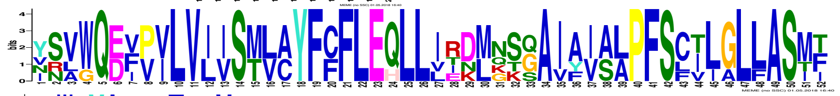
	(7)	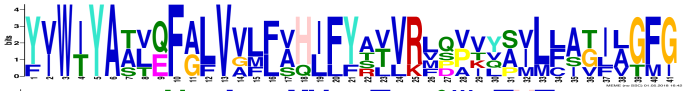
Fv05	(8)	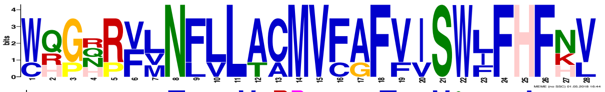
Fv06	(14)	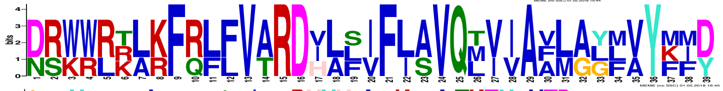
	(9)	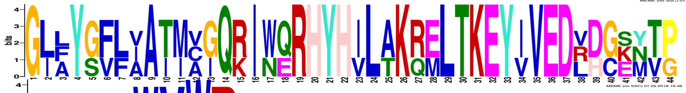
Lv02	(42)	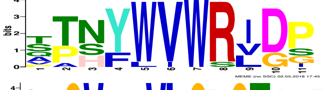
	(34)	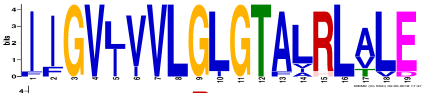
	(43)	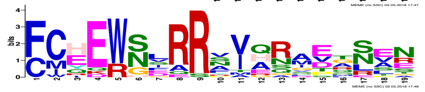

RING-D	LOGO#	LOGO sequence
FD	(2)	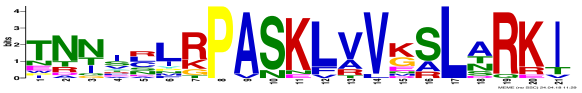
	(3)	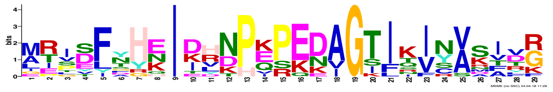
	(4)	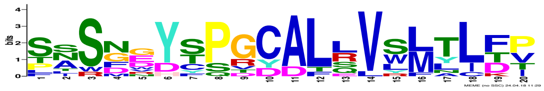

RING-G	LOGO#	LOGO sequence
FG	(5)	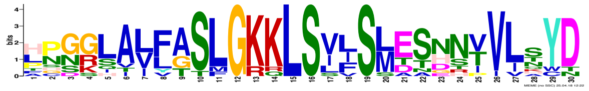
	(9)	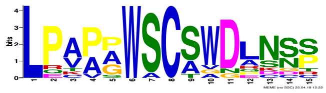
	(1)	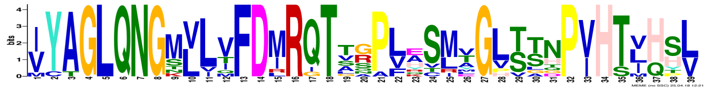

RING-S/T	LOGO#	LOGO sequence
FS/T	(4)	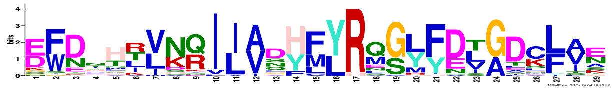
	(3)	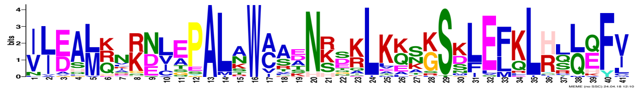
	(2)	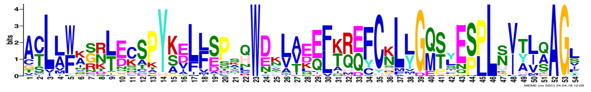
